# A Cotton Laccase Confers Disease Resistance Against *Verticillium dahliae* by Promoting Cell Wall Lignification

**DOI:** 10.1111/mpp.70125

**Published:** 2025-07-14

**Authors:** Guanfu Cheng, Chuanzong Li, Guoshuai Zhang, W. G. Dilantha Fernando, Yanqing Bi, Jianfeng Lei, Peihong Dai, Xiaofeng Su, Yue Li

**Affiliations:** ^1^ Xinjiang Key Laboratory for Ecological Adaptation and Evolution of Extreme Environment Biology, College of Life Sciences Xinjiang Agricultural University Xinjiang China; ^2^ National Key Laboratory of Agricultural Microbiology, Biotechnology Research Institute Chinese Academy of Agricultural Sciences Beijing China; ^3^ Department of Plant Sciences University of Manitoba Winnipeg Manitoba Canada

**Keywords:** cotton disease resistance, *GhLAC14‐3*, *GhMAPKKK2*, lignin synthesis, Verticillium wilt

## Abstract

Verticillium wilt (VW), caused primarily by *Verticillium dahliae*, is a significant threat to cotton production. Lignification of the plant cell wall, a defence response triggered by pathogen invasion, is critical for plant resistance to numerous diseases. Laccases are known to participate in the lignification of secondary cell walls, but their role in cotton resistance to *V. dahliae* is not fully understood. In this study, we identified a cotton laccase gene, *GhLAC14‐3*, that was significantly upregulated during early *V. dahliae* infection and was closely related to a gene previously reported to respond to *V. dahliae* infection in *Arabidopsis*. Silencing of *GhLAC14‐3* in cotton increased disease susceptibility and reduced lignin deposition and the expression of lignin‐related genes. By contrast, overexpression of *GhLAC14‐3* in transgenic *Arabidopsis* increased lignin content and the expression of lignin‐related genes, thereby enhancing VW resistance. We identified an interaction between GhLAC14‐3 and the mitogen‐activated protein kinase GhMAPKKK2 at the cell membrane. *GhMAPKKK2* expression was also significantly induced by *V. dahliae* infection in cotton, and its overexpression in *Arabidopsis* activated multiple key resistance genes, thus improving *V. dahliae* resistance*.* Transient co‐expression of *GhMAPKKK2* and *GhLAC14‐3* in *Nicotiana benthamiana* leaves significantly increased lignin content. Conversely, silencing of *AtMAPKKK2*, the homologue of *GhMAPKKK2*, in *GhLAC14‐3*‐overexpressing *Arabidopsis* reduced both lignin levels and disease resistance. Our findings suggest that *Gh*LAC14‐3 is a promising target for enhancing VW resistance, as its interaction with *Gh*MAPKKK2 at the cell membrane modulates defence‐induced lignification.

## Graphical abstract

Upon *V. dahliae* infection, cotton enhances resistance by upregulating lignin synthesis genes and oxidising lignin monomers via laccase *Gh*LAC14‐3 and *Gh*MAPKKK2 to form lignin and lignify cell walls.

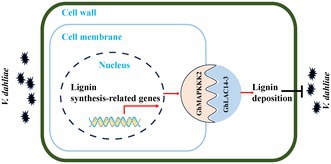

## 1 Introduction

Cotton (*Gossypium* spp.) is a globally significant economic crop, valued for its natural fibres, edible oils, and proteins (Adeleke 2024). However, cotton production is severely impacted by various biotic and abiotic stresses that compromise yield and quality (Billah et al. 2021). One of the most significant threats is Verticillium wilt (VW), a vascular disease caused by the soil‐borne fungus *Verticillium dahliae* (Zhang et al. 2022), which affects over 200 plant species and leads to substantial agricultural losses (Song et al. 2020; Zhu et al. 2023; Umer et al. 2023). *V. dahliae* typically germinates from dormant microsclerotia, with hyphae that invade cotton roots and spread systemically, manifesting as leaf wilting, chlorosis, browning of microtubules and ultimately leading to plant death. Under unfavourable conditions, it forms microsclerotia that persist in the soil, maintaining virulence for extended periods (Ma et al. 2021; Shaban et al. 2018; Wang et al. 2022; Dhar et al. 2020). Upland cotton ( *Gossypium hirsutum* ), which constitutes about 90% of global cotton production, is particularly vulnerable to VW (Zhu et al. 2023). Despite its prevalence, only a few resistant germplasms and resistance genes have been identified, complicating VW management and prevention (Klosterman et al. 2009). Thus, discovering new resistance genes and developing resistant cultivars are crucial for effectively combating VW (Li, Zhang, et al. 2021).

Plants employ a range of complex defence mechanisms to resist *V. dahliae* infection, including cell wall strengthening and remodelling, accumulation of secondary metabolites and activation of defence genes (Zhou and Zhang 2020; Ngou, Ding, and Jones 2022; Shaban et al. 2018; Dhar et al. 2020). The cell wall, primarily composed of lignin, serves as the primary barrier against pathogen invasion and is crucial for disease resistance (Bacete et al. 2018; Narváez‐Barragán et al. 2022). Lignin enhances mechanical strength and plays a key role in plant growth, development, and stress responses (Kozieł et al. 2021; Ishida and Noutoshi 2022). The phenylpropanoid pathway, a major secondary metabolic pathway in plants, is essential for defence against biotic and abiotic stresses (La Camera et al. 2004). Lignin synthesis, a critical branch of this pathway, involves the polymerisation of monolignols, reinforcing the cell wall and forming a robust barrier that restricts pathogen entry (Bonello and Blodgett 2004).

Lignin deposition in infected cells plays a multifaceted defensive role in cotton. It not only restricts pathogen entry and limits toxin and enzyme production but also impedes the transfer of water and nutrients from the plant to the pathogen, thereby suppressing growth (Ma 2024). Research has shown that the cotton cultivar *Gossypium barbadense* 7124, resistant to *V. dahliae*, exhibits higher lignin content and upregulated lignin biosynthesis genes compared to the susceptible *G. hirsutum* YZ‐1 (Xu et al. 2011; Yang et al. 2021). Various lignin biosynthesis‐related genes are critical for VW resistance, such as laccases *GhLAC1* and *GhLAC15*, cinnamyl alcohol dehydrogenase genes *GhCAD35*, *GhCAD43*, *GhCAD45* and the 4‐coumarate‐CoA ligase genes *Gh4CL3*, *Gh4CL30*, along with the ferulate 5‐hydroxylase gene *GhF5H* (Zhang et al. 2019; Li, Zhang, Zhao, et al. 2022; Xiong et al. 2021; Alariqi et al. 2023; Wei et al. 2024; Hu et al. 2018). Transcription factors like MYB, WRKY and the ethylene‐response factor *Gb*ERF1‐like from *G. barbadense* also modulate lignin accumulation and VW resistance, enhancing disease tolerance through various pathways (Xiao et al. 2021). Moreover, melatonin and the histone deacetylase *Gh*HDA5 influence VW resistance by regulating lignin biosynthesis genes (Li et al. 2019; Wei et al. 2024). These findings underscore the crucial role of lignin in cotton resistance to VW, highlighting the complexity of its regulatory network and providing a foundation for targeted genetic and molecular improvements in cotton disease resistance.

Laccases, part of the blue copper oxidase protein family, play a critical role in plant pathogen resistance by promoting lignin synthesis across various species (Cai et al. 2006; Bai et al. 2023). For example, in *Arabidopsis thaliana* , LACCASE2 negatively regulates lignin deposition, with its mutation increasing lignin content in root vascular tissues (Khandal et al. 2020). In cotton, overexpressing *GhLAC1* enhances lignification and balances jasmonic acid defences, boosting resistance to *V. dahliae* (Hu et al. 2018). Similarly, *GhLAC15* enhances resistance in transgenic *Arabidopsis* by increasing lignin content and altering the syringyl (S) to guaiacyl (G) ratio; in contrast, its silencing in cotton heightens susceptibility to pathogens (Zhang et al. 2019). In apple ( *Malus domestica* ), increased lignification activates defences against *Alternaria alternata* through *Md*LAC7, mediated by *Md*WRKY75e (Zhu et al. 2021; Hou et al. 2021). Moreover, overexpression of *LAC* thickens xylem walls, enhances resistance to *Botrytis cinerea* in tobacco, and improves resistance to *A. alternata* and *Botryosphaeria* in pear (Zhao et al. 2022; Yang et al. 2023).

MAPK kinases interact with and phosphorylate downstream target proteins, thereby altering their activity (Zhang et al. 2022). In *Arabidopsis*, MPK3/6 interacts with PIF3 (phytochrome‐interacting factor 3), leading to phosphorylation that upregulates defence genes and enhances resistance to *Pseudomonas syringae* pv. *tomato* (Zhao et al. 2023). In rice, MKP1 phosphorylates the MYB4 transcription factor, which negatively regulates vascular lignification by inhibiting lignin biosynthesis (Lin et al. 2022). *Os*MPK1 interacts with *Os*BBX17 and phosphorylates it at the Thr‐95 site, which reduces the DNA‐binding activity of *Os*BBX17 and enhances saline‐alkaline tolerance by deregulating transcriptional repression of *OsHAK2* and *OsHAK7* (Shen et al. 2024). *Os*MAPK3 phosphorylates *Os*NAC29 at Thr‐304 to prevent its proteasome‐mediated degradation and promote its function against the rice blast fungus (Lu et al. 2024). In cotton, *Gh*WRKY40a phosphorylated by *Gh*MPK9 can activate the transcription of *GhERF1b* to upregulate defence‐related genes and inhibit the transcription of *GhABF2* to regulate stomatal opening, thus improving resistance to VW (Mi et al. 2024).

This study aimed to explore the molecular mechanisms underlying cotton resistance to *V. dahliae*. By analysing transcriptomic data from *Arabidopsis thaliana* roots inoculated with *V. dahliae* (Su et al. 2018), we found that the laccase gene *AtLAC14‐3* was significantly upregulated during infection. We then characterised its cotton ( *G. hirsutum* ) homologue, *GhLAC14‐3*, which was notably upregulated in response to the pathogen. Through virus‐induced gene silencing (VIGS) and heterologous expression in *Arabidopsis*, we confirmed that *GhLAC14‐3* enhances plant resistance to *V. dahliae* by promoting lignin biosynthesis. Further yeast two‐hybrid (Y2H) screening revealed an interaction between *Gh*LAC14‐3 and *Gh*MAPKKK2, which was confirmed by bimolecular fluorescence complementation (BiFC) and luciferase complementation imaging (LCI) assays. Overexpression of *GhMAPKKK2* in *Arabidopsis* activated defence responses and significantly improved resistance to VW. Transient co‐expression of *GhMAPKKK2* and *GhLAC14‐3* in *Nicotiana benthamiana* led to a significant increase in lignin levels, whereas downregulation of *AtMAPKKK2* in *GhLAC14‐3*‐overexpressing *Arabidopsis* plants reduced lignin levels and disease resistance. These findings suggest that *GhLAC14‐3* enhances cotton resistance to VW by promoting lignin accumulation. Moreover, *Gh*MAPKKK2 likely functions as an upstream regulator to activate *Gh*LAC14‐3, further enhancing resistance to VW. Overall, this research highlights the critical roles of *Gh*LAC14‐3 and *Gh*MAPKKK2 in cotton's defence against VW and provides new insights into the regulatory mechanisms of lignin metabolism and disease resistance.

## 2 Results

### 2.1 *GhLAC14‐3* Expression Is Responsive to *V. dahliae* Infection

*Gh*LAC14‐3 contains a laccase superfamily domain, which was identified through Conserved Domains Search on the NCBI website (https://www.ncbi.nlm.nih.gov/Structure/cdd/wrpsb.cgi) (Figure S1a). It shows close evolutionary relationships with homologous cotton proteins, including *Gh*LAC14‐1 (Figure S1b). The predicted tertiary structure of *Gh*LAC14‐3 includes five potential phosphorylation sites (S53, S72, T28, T171 and T193) that may be recognised by kinases (Figure S1c). BlastP analysis revealed that *Gh*LAC14‐3 from *G. hirsutum* is highly homologous to laccase proteins from *Gossypium arboretum* (Figure S1d).

**FIGURE 1:**
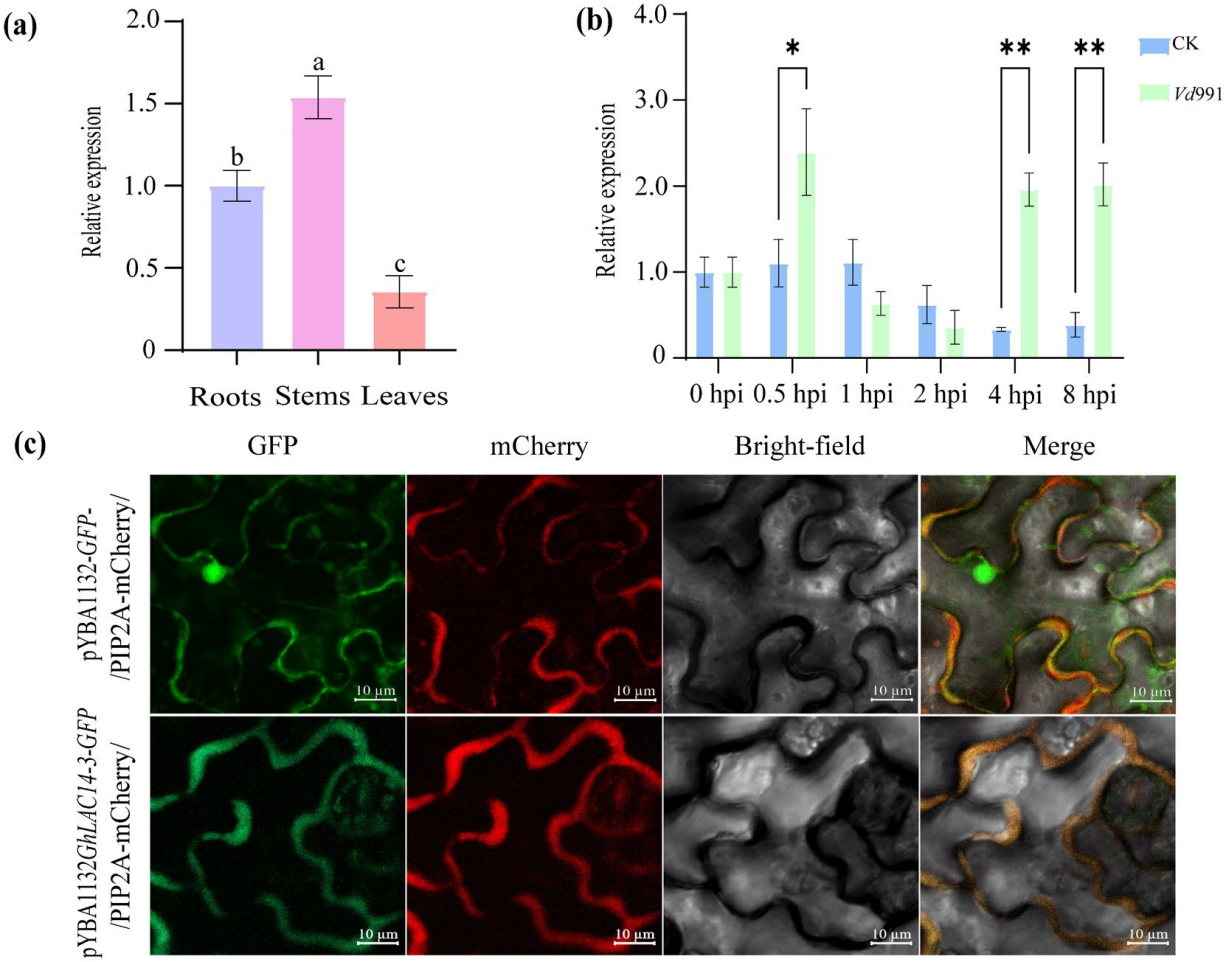
Expression and subcellular localisation analysis of *Gh*LAC14‐3. (a) Tissue‐specific expression of *GhLAC14‐3* in cotton roots, stems, and leaves. Data represent means ± SD from three biological replicates. Statistical significance was determined by one‐way ANOVA followed by Duncan's test. Different letters denote significant differences (*p* ≤ 0.05) among groups. (b) Expression of *GhLAC14‐3* during *Verticillium dahliae* infection. *GhLAC14‐3* expression was analysed in cotton roots at different time points post‐infection with *V. dahliae* (hours post‐inoculation, hpi). Data are presented as means ± SD from three biological replicates, with three plants per replicate. CK, negative control. Significant differences were determined using Student's *t* test (*p < 0.05, **p < 0.01). (c) Subcellular localisation of *Gh*LAC14‐3‐GFP in *Nicotiana benthamiana* leaves. Nuclear localisation was confirmed with H2B‐mCherry, and membrane localisation was confirmed with PIP2A‐mCherry. Free GFP was used as a control. Scale bars = 10 μm.

Tissue‐specific expression analysis indicated that *GhLAC14‐3* is ubiquitously expressed across all tissues, with relatively higher expression levels observed in stems, suggesting a potential role in stem xylem development (Figure 1a). Further, we analysed the expression pattern of *GhLAC14‐3* in response to *V. dahliae* infection. Reverse transcription‐quantitative PCR (RT‐qPCR) analysis revealed that the expression levels of *GhLAC14‐3* were significantly upregulated at 0.5, 4 and 8 h post‐inoculation (hpi) (Figure 1b). These findings suggest that *GhLAC14‐3* may play a role in the cotton defence response to *V. dahliae*.

To further elucidate the functional role of *GhLAC14‐3*, we investigated its subcellular localisation. We transiently expressed the *Gh*LAC14‐3‐GFP fusion protein in *N. benthamiana* leaves through *Agrobacterium tumefaciens* ‐mediated infiltration. Subsequent observations using a laser‐scanning confocal microscopy revealed that the *Gh*LAC14‐3‐GFP fusion protein exclusively co‐localised with PIP2A‐mCherry to the plasma membrane (Figure 1c). In contrast, free GFP exhibited widespread distribution throughout the cell, including the cell membrane, cytoplasm, and nucleus. These results suggest that *Gh*LAC14‐3 is specifically localised to the cell membrane.

### 2.2 *GhLAC14‐3* Positively Regulates Cotton Resistance to *V. dahliae* Infection

**FIGURE 2:**
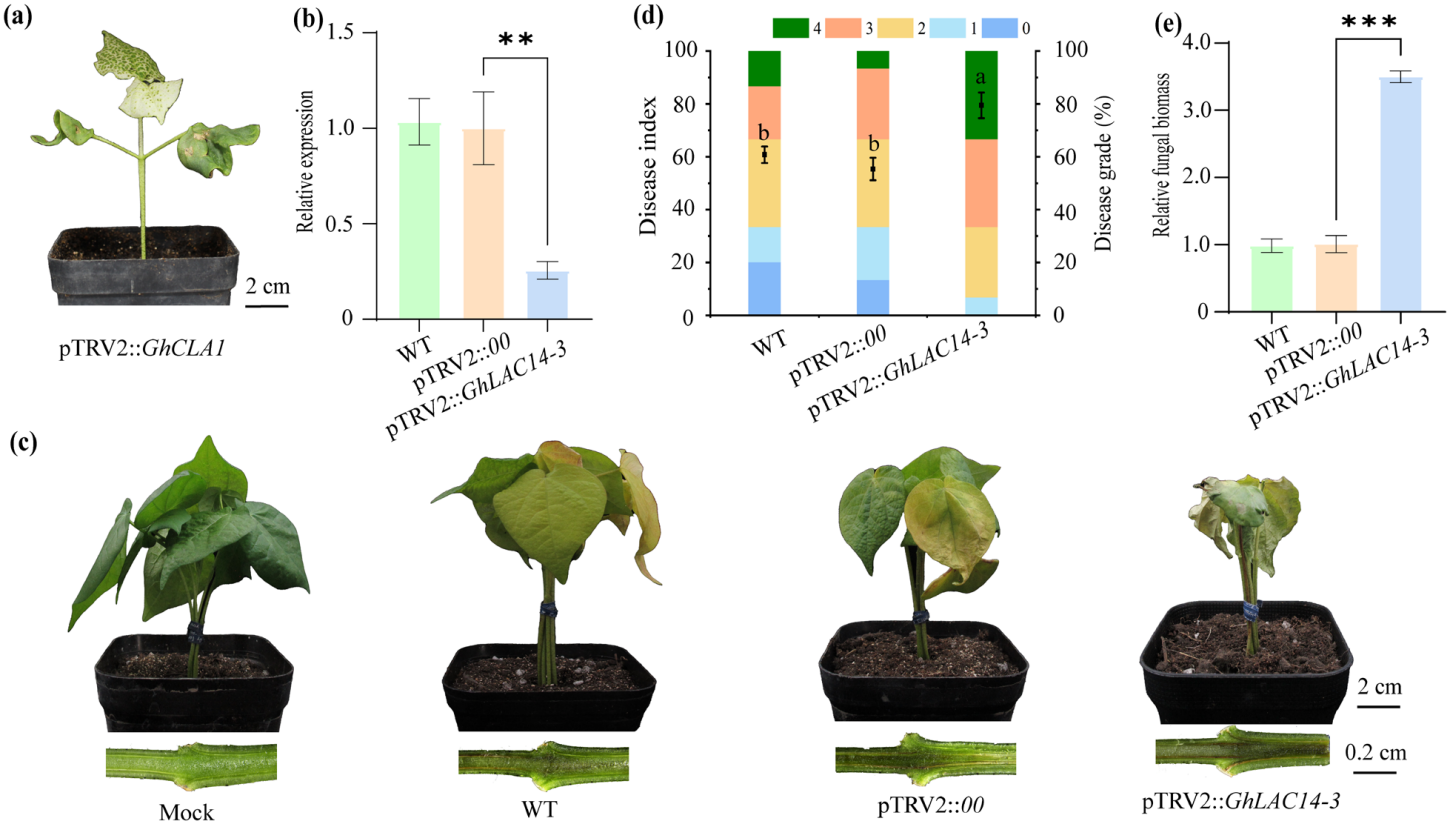
Silencing of *GhLAC14‐3* reduces *Verticillium dahliae* resistance in *Gossypium hirsutum* . (a) Leaf phenotype of a cotton plant with silenced *GhCLA1*, confirming the effectiveness of the virus‐induced gene silencing (VIGS) system. (b) Relative expression levels of *GhLAC14‐3* in wild‐type (WT), pTRV2::00, and pTRV2::*GhLAC14‐3* plants. (c–e) Disease symptoms, (c) disease indexes (d), and fungal biomass (e) of WT, pTRV2::00, and pTRV2::*GhLAC14‐3* plants at 14 days post‐inoculation. Cotton plants were grown in a growth chamber maintained at 28°C, with 75% relative humidity, a light intensity of 400 μmol m^−2^ s^−1^, and a photoperiod of 16 h of light and 8 h of darkness. Data in (b) and (c) represent the means ± SE from three biological replicates, each containing five plants per genotype. Asterisks indicate statistically significant differences determined by Student's *t* test (**p < 0.01, ***p < 0.001). Values in (d) are the means ± SE from three biological replicates, each containing 10 plants per genotype. Statistical significance was determined using one‐way ANOVA followed by Duncan's test. Different letters denote significant differences (*p* ≤ 0.05) among groups.

To investigate the role of *GhLAC14‐3* in cotton defence against VW, we knocked down *GhLAC14‐3* in cotton using a VIGS system. Two weeks after *Agrobacterium* infiltration, wild‐type (WT), pTRV2::00 (the empty vector control) and pTRV2::*GhLAC14‐3* (the VIGS vector carrying the gene construct for silencing *GhLAC14‐3*) plants were inoculated with *V. dahliae*. Photobleaching was observed on newly emerged leaves of the seedlings transformed with pTRV2::*GhCLA1*, indicating successful gene silencing (Figure 2a). Silencing was further confirmed by RT‐qPCR, which revealed a 65% reduction in *GhLAC14‐3* expression (Figure 2b). The pTRV2::*GhLAC14‐3* plants displayed more pronounced VW symptoms than control plants, including wilted leaves and browned vascular bundles (Figure 2c), as well as a significantly higher disease index and greater disease severity (Figure 2d). Fungal biomass was 3.5‐fold higher in *GhLAC14‐3*‐silenced than in control plants (Figure 2e), further supporting the notion that disruption of *GhLAC14‐3* function compromises cotton resistance to *V. dahliae*.

### 2.3 Silencing of *GhLAC14‐3* Reduces Lignin Deposition

**FIGURE 3:**
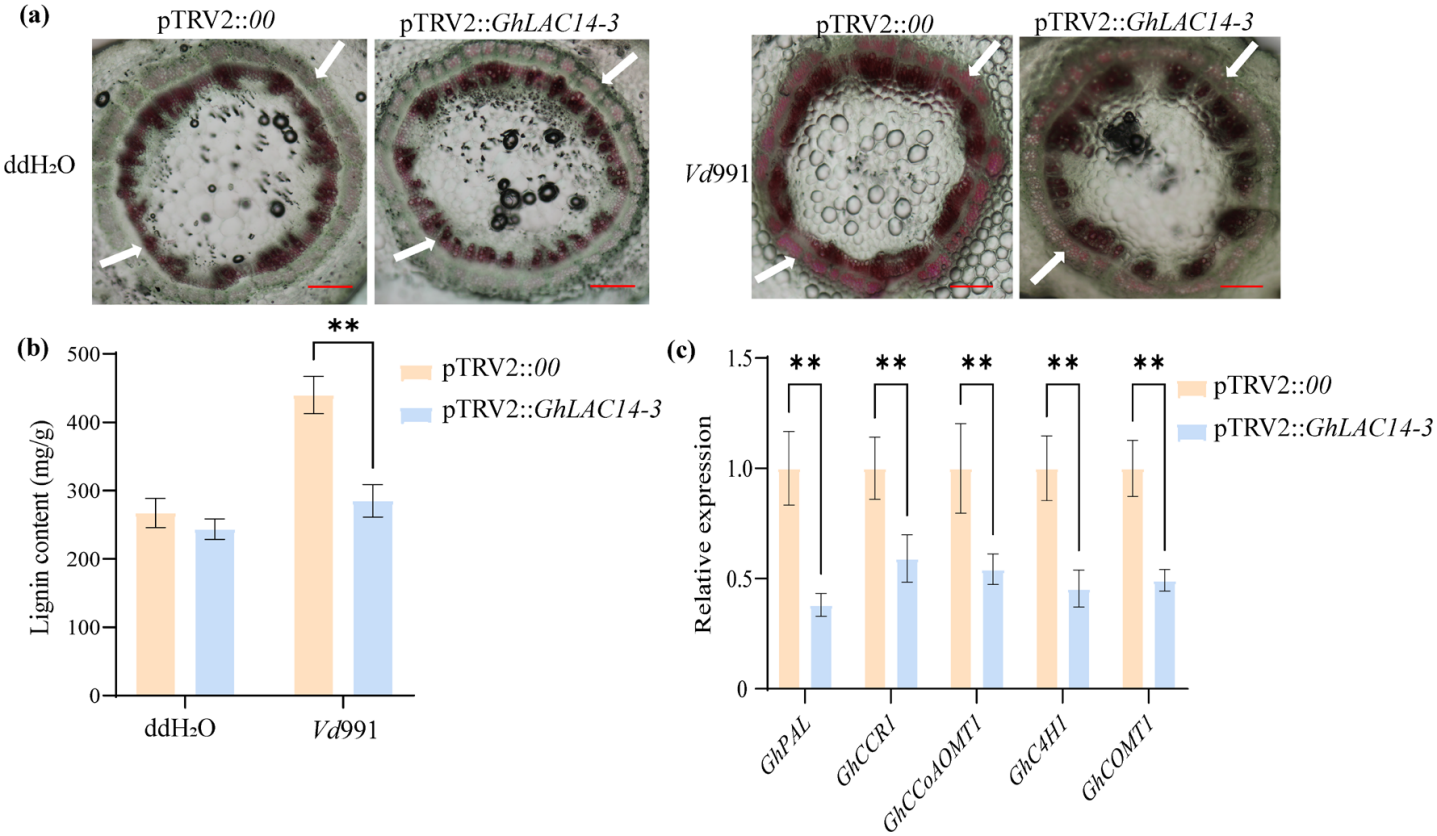
Silencing of *GhLAC14‐3* reduces lignin deposition in *Gossypium hirsutum* . (a) Histochemical staining of lignin deposition in cross‐sections of pTRV2::00 and pTRV2::*GhLAC14‐3* stems at 2 h post‐inoculation (hpi) with water or *Verticillium dahliae*. Scale bars = 0.05 cm. Cotton plants were grown in a chamber set at 28°C, with 75% relative humidity, a light intensity of 400 μmol m^−2^ s^−1^, and a 16 h light/8 h dark photoperiod. (b) Quantification of lignin content in the roots of pTRV2::00 and pTRV2::*GhLAC14‐3* plants at 2 hpi with water or *V. dahliae*. (c) Relative expression levels of five lignin biosynthesis‐related genes (*GhPAL*, *GhCCR1*, *GhCCoAOMT1*, *GhC4H1*, and *GhCOMT1*) in pTRV2::00 and pTRV2::*GhLAC14‐3* plants at 2 hpi with *V. dahliae*. *GhUBQ7* (LOC107912293) served as the endogenous control. Data are presented as means ± SE from three biological replicates; within each biological replicate, the entire primary roots from three plants per genotype were analysed. Asterisks indicate statistically significant differences determined by Student's *t* test (**p < 0.01).

To determine the role of *GhLAC14‐3* in cotton resistance to VW via its impact on lignin biosynthesis, we performed histochemical staining and quantified lignin content in pTRV2::00 (control) and pTRV2::*GhLAC14‐3* (*GhLAC14‐3*‐silenced) cotton plants. Under mock conditions, no visible differences in lignin accumulation were observed between *GhLAC14‐3*‐silenced plants and the pTRV2::00 control. However, after inoculation with *Vd*991, both groups exhibited increased lignin deposition compared to water‐treated controls. Notably, *GhLAC14‐3*‐silenced plants showed significantly lower lignin accumulation compared to pTRV2::00 plants following the treatment (Figure 3a,b). Further molecular analysis revealed decreased expression of five key lignin biosynthesis marker genes: *GhPAL*, *GhCCR1*, *GhCCoAOMT1*, *GhC4H1* and *GhCOMT1* in *GhLAC14‐3*‐silenced plants compared to controls (Figure 3c). These findings suggest that the silencing of *GhLAC14‐3* impairs lignin deposition, correlating with the downregulation of lignin biosynthesis genes. This evidence supports the role of *GhLAC14‐3* in promoting lignin accumulation and underscores its importance in lignin‐mediated defence against VW.

### 2.4 Heterologous Overexpression of *GhLAC14‐3* Enhances *Arabidopsis* Resistance to VW

**FIGURE 4:**
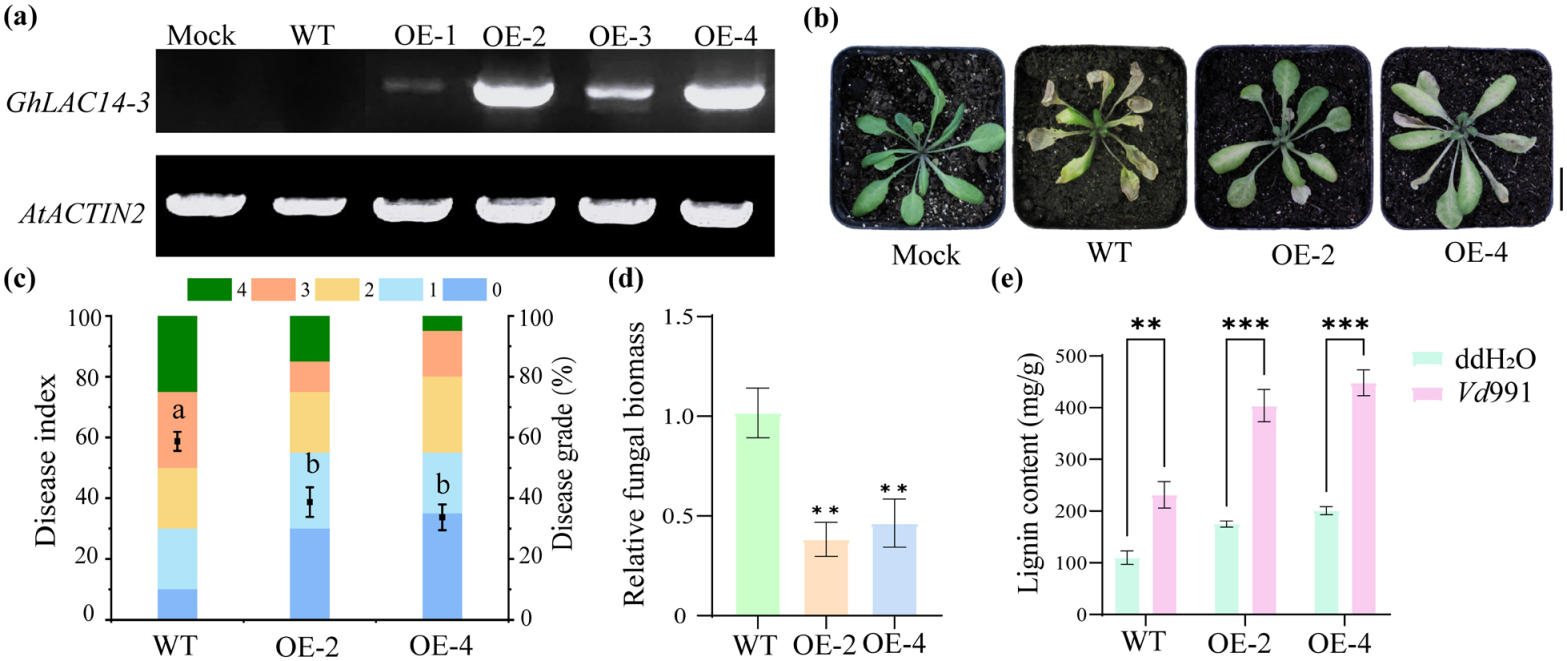
Heterologous expression of *GhLAC14‐3* enhances *Verticillium dahliae* resistance in *Arabidopsis*. (a) Expression level of *GhLAC14‐3* in wild‐type (WT) and *GhLAC14‐3* overexpression (*GhLAC14‐3*‐OE) plants, normalised to *AtActin2*. (b–e) Analysis of disease symptoms, (b) disease indices, (c) fungal biomass, and (e) lignin content in WT and *GhLAC14‐3* overexpression plants at 2 h post‐inoculation (hpi) with *V. dahliae*. (c) Disease index values are presented as means ± SE from three biological replicates, each containing 10 plants per genotype. Plants were grown in a chamber set at 28°C, with 75% relative humidity, a light intensity of 400 μmol m^−2^ s^−1^, and a 16 h light/8 h dark photoperiod. Statistical significance was determined using one‐way ANOVA followed by Duncan's test. Different letters denote no significant differences (*p* ≤ 0.05) among groups. (d, e) Fungal biomass and lignin content are shown as means ± SE from three biological replicates, with plants per genotype. Statistical significance was determined using Student's *t* test (**p < 0.01, ***p < 0.001).

To further elucidate the role of *GhLAC14‐3* in plant resistance to *V. dahliae*, we transformed *Arabidopsis thaliana* seedlings with *GhLAC14‐3* driven by the 35S promoter via *A. tumefaciens* ‐mediated transformation (Figure S2). RT‐qPCR analysis confirmed significantly elevated *GhLAC14‐3* expression in transgenic lines *GhLAC14‐3‐*OE #2 and *GhLAC14‐3‐*OE #4, which were selected for subsequent experiments (Figure 4a). At 14 days post‐inoculation (dpi) with *V. dahliae*, transgenic plants exhibited substantially reduced disease symptoms compared to WT plants, including less wilting and chlorosis, lower disease index and reduced fungal biomass (Figure 4b–d). Additionally, lignin content analysis revealed increased deposition in *V. dahliae*‐inoculated plants, with transgenic plants showing significantly higher lignin levels than WT (Figure 4e). Notably, key lignin biosynthesis‐related genes *AtNST1*, *AtPRN2*, *AtWakl8*, *AtMYB15* and *AtSHMT6* were significantly upregulated in *GhLAC14‐3‐*OE compared with WT plants with *V. dahliae* inoculation, when using *Actin2* (AT3G18780) as the internal reference (Figure S3). These results collectively demonstrate that ectopic expression of *GhLAC14‐3* enhances *Arabidopsis* resistance to *V. dahliae*, likely through increased lignin biosynthesis and the upregulation of lignin‐associated genes, thereby strengthening defences against this pervasive vascular pathogen.

### 2.5 *Gh*LAC14‐3 Interacts With *Gh*MAPKKK2

**FIGURE 5:**
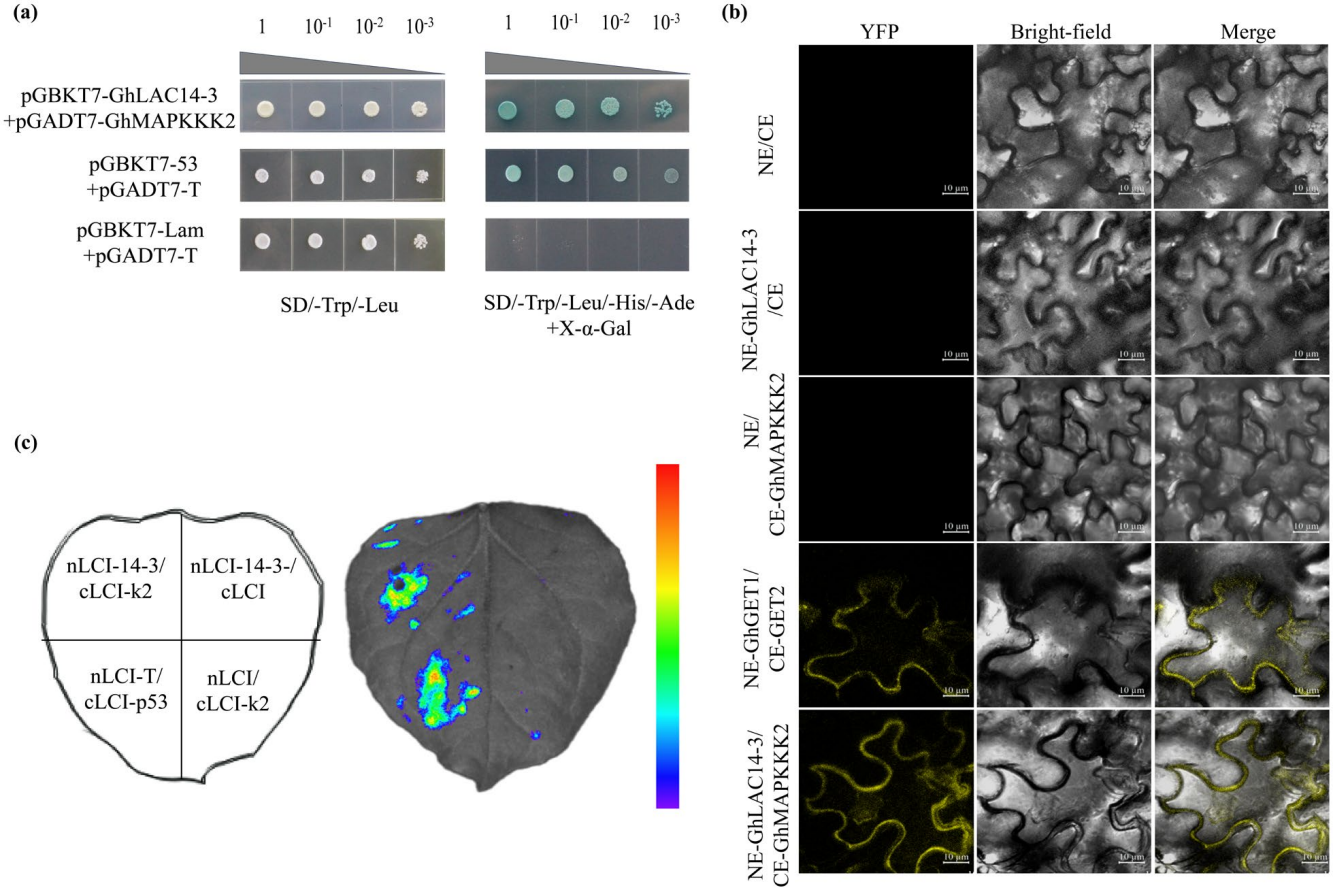
Interactions between *Gh*LAC14‐3 and *Gh*MAPKKK2. (a) Yeast two‐hybrid (Y2H) assay showing interactions between *Gh*LAC14‐3 and *Gh*MAPKKK2. BD‐p53/AD‐T and BD‐Lam/AD‐T served as positive and negative controls, respectively. SD/−Trp−Leu indicates a synthetic defined medium lacking leucine and tryptophan; SD/−Trp−Leu−His−Ade indicates a medium lacking leucine, tryptophan, histidine, and adenine; X‐α‐gal refers to 5‐bromo‐4‐chloro‐3‐indoxyl‐α‐d‐galactopyranoside. (b) Bimolecular fluorescence complementation assay showing interactions between *Gh*LAC14‐3 and *Gh*MAPKKK2. Scale bars: 10 μm. (c) Luciferase complementation imaging assay confirming the interaction between *Gh*LAC14‐3 and *Gh*MAPKKK2.

To elucidate the regulatory mechanism by which *Gh*LAC14‐3 enhances cotton resistance to *V. dahliae*, we cloned the coding sequence (CDS) of *GhLAC14‐3* into the pGBKT7 (BD) vector to construct a bait plasmid (BD‐*Gh*LAC14‐3). Through Y2H library screening, we identified a protein that interacts with *Gh*LAC14‐3, which we designated as *Gh*MAPKKK2 due to its MAPKKK structural domain (Figure 5a). This interaction was further validated in vivo using BiFC and LCI assays. In the BiFC assay, co‐expression of *GhLAC14‐3*‐nYFP and *GhMAPKKK2*‐cYFP in *N. benthamiana* resulted in YFP fluorescence localised at the cell membrane, whereas no fluorescence was observed in the control combinations, including *GhLAC14‐3*‐nYFP with cYFP, nYFP with *GhMAPKKK2*‐cYFP or cYFP with nYFP (Figure 5b). Similarly, in the LCI assay, visible fluorescence was detected on the leaf surface when *GhLAC14‐3*‐nLUC and *GhMAPKKK2*‐cLUC were co‐expressed in *N. benthamiana*. In contrast, no signal was observed in control groups, including *GhLAC14‐3*‐nLUC co‐expressed with cLUC or nLUC co‐expressed with *GhMAPKKK2*‐cLUC (Figure 5c). These results confirm that *Gh*LAC14‐3 specifically interacts with *Gh*MAPKKK2 and that this interaction occurs at the cell membrane.

### 2.6 *GhMAPKKK2* Contributes to Plant Resistance Against *V. dahliae*

Analysis of conserved structural domains and motifs using NCBI tools revealed that *Gh*MAPKKK2 contains a characteristic serine/threonine kinase catalytic domain (Figure S4a). Further predictions of its secondary and tertiary structures identified the location of its active site (Figure S4b–c). A BlastP search against the NCBI database showed that *Gh*MAPKKK2 shares high homology with MAPKKK proteins from other species, particularly *G. arboreum* (Figure S4d). Expression analysis indicated that *GhMAPKKK2* is present in cotton roots, stems, and leaves, with notably higher expression in stems and leaves compared to roots (Figure S5a). Upon *V. dahliae* infection, *GhMAPKKK2* exhibited a similar expression pattern to *GhLAC14‐3*, with significant upregulation at 0.5, 4, and 8 hpi compared to untreated controls (Figure S5b). Subcellular localisation studies were conducted by transiently expressing a *GhMAPKKK2*‐GFP fusion protein in *N. benthamiana* leaves. The fluorescence signals from *GhMAPKKK2*‐GFP exclusively co‐localised with the membrane marker PIP2A‐mCherry at the cell membrane (Figure S5c). In contrast, free GFP signals were distributed throughout the cell. Taken together, these findings suggest that *Gh*MAPKKK2 is a membrane‐localised protein potentially involved in early responses to *V. dahliae* infection.

**FIGURE 6:**
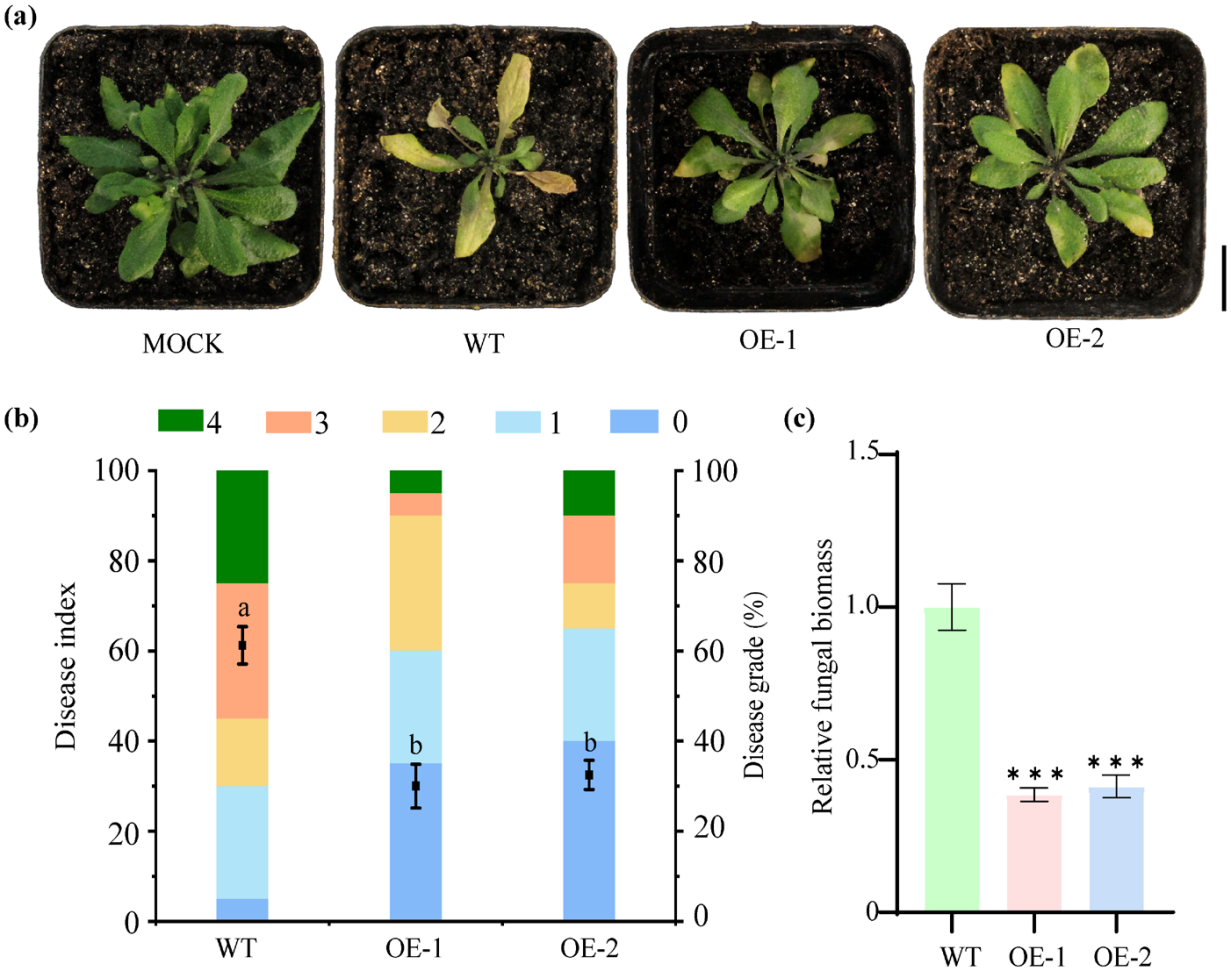
Overexpression of *GhMAPKKK2* enhances resistance to *Verticillium dahliae* in *Arabidopsis*. (a) Phenotypes of wild‐type (WT) and *GhMAPKKK2*‐OE (overexpressing) plants following *V. dahliae* inoculation at 14 days post‐inoculation (dpi). At least 20 2‐week‐old plants from each line were inoculated, replanted in soil, and photographed at 14 dpi. Plants were grown in a growth chamber set at 28°C, with 75% relative humidity, a light intensity of 400 μmol m^−2^ s^−1^, and a 16 h light/8 h dark photoperiod. Representative images are shown. Scale bars = 2 cm. (b) Disease index values are presented as means ± SE from three biological replicates, each consisting of 10 plants. Statistical significance was determined using one‐way ANOVA followed by Duncan's test. Different letters denote no significant differences (*p* < 0.05) among groups. (c) Fungal biomass is shown as means ± SE from three biological replicates, with five plants per genotype. Statistical significance was determined using Student's *t* test (***p < 0.001).

To investigate whether *GhMAPKKK2* contributes to plant defence against *V. dahliae*, we overexpressed the gene in *Arabidopsis* seedlings using *A. tumefaciens* ‐mediated transformation. The transgenic lines exhibited significantly elevated levels of *GhMAPKKK2* expression compared to WT controls (Figure S5d). Two weeks post‐inoculation with the *V. dahliae* Vd991, these *GhMAPKKK2*‐OE lines demonstrated much milder symptoms of wilt disease than control plants, including reduced leaf wilting and necrosis, lower disease index values and decreased fungal biomass (Figure 6). Additionally, we observed a significant upregulation of five defence‐related genes (*AtNDR1*, *AtRIN4*, *AtRPM1*, *AtMIR399*, *AtRPW8* and *AtJAV1*) in *GhMAPKKK2*‐OE lines (Dhar et al. 2019; Zhang et al. 2023; Prokchorchik et al. 2020; Saile et al. 2021; Xiao et al. 2004) (Figure S5e). These findings collectively suggest that *GhMAPKKK2* not only enhances resistance but also modulates critical pathways involved in combating *V. dahliae*. Specifically, our results indicate that *GhMAPKKK2* overexpression enhances *Arabidopsis* resistance to *V. dahliae*.

### 2.7 The Interaction Between *Gh*MAPKKK2 and *Gh*LAC14‐3 Is Essential for Enhanced Lignin Production Mediated by *Gh*LAC14‐3

**FIGURE 7:**
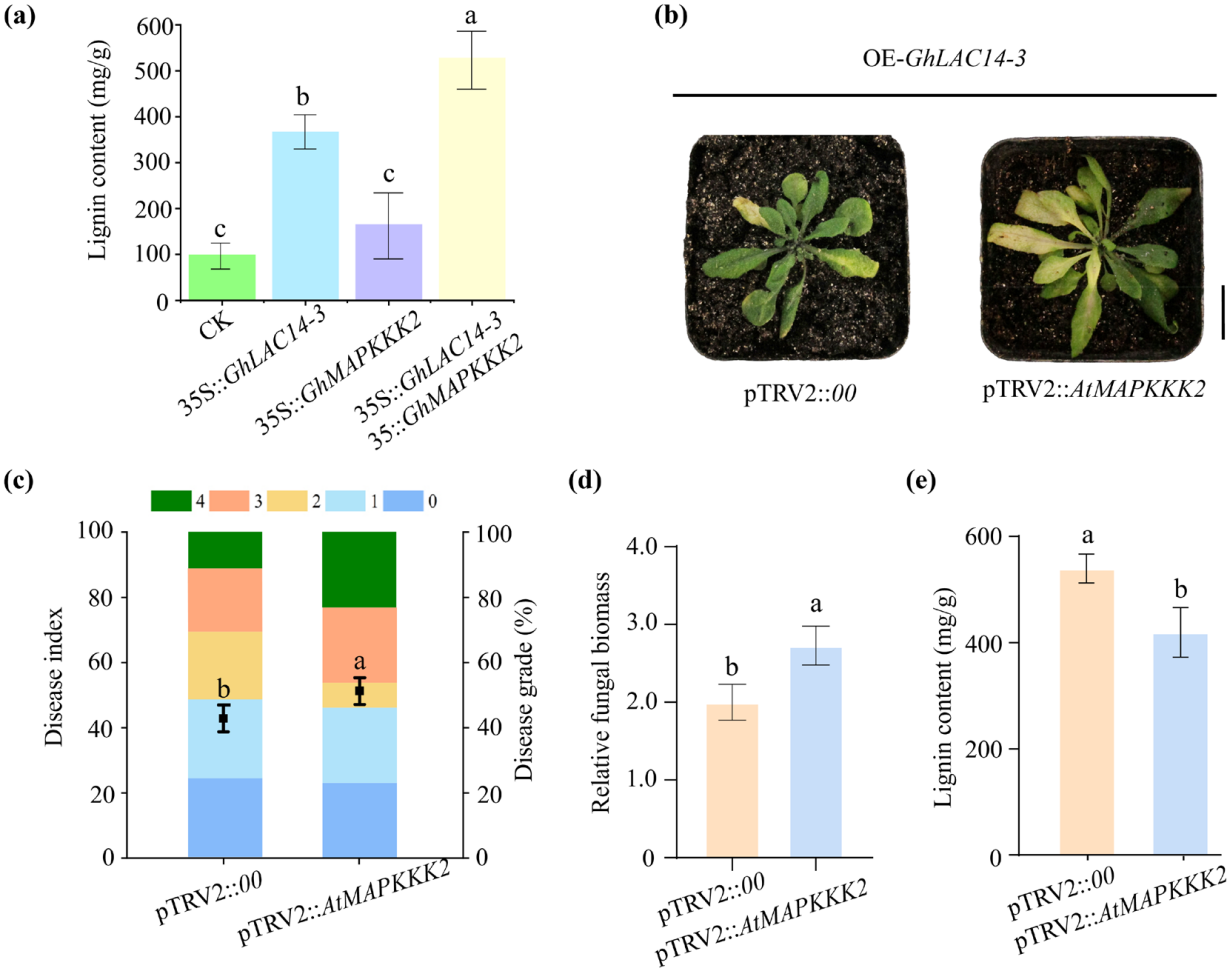
*Gh*MAPKKK2 interacts with *Gh*LAC14‐3 to regulate lignin biosynthesis and enhance disease resistance. (a) Lignin content in *Nicotiana benthamiana* leaves 48 h after co‐infiltration with equal amounts of *Agrobacterium* cells harbouring different plasmids. (b) Disease symptoms in *Arabidopsis* at 14 days post‐inoculation. (c) Disease index. Values are presented as means ± SE from three biological replicates, each genotype tested consisting of 10 plants. (d) Relative fungal biomass. Data are presented as means ± SE from three biological replicates, each genotype tested consisting of five plants. (e) Lignin content in OE‐*GhLAC14‐3* and pTRV2::*AtMAPKKK2*. Plants were grown in a growth chamber set at 28°C, with 75% relative humidity, a light intensity of 400 μmol m^−2^ s^−1^, and a 16 h light/8 h dark photoperiod. Data are shown as means ± SE from three biological replicates, with five plants per genotype. Statistical significance was determined using one‐way ANOVA followed by Duncan's test. Different letters denote no significant differences (*p* < 0.05) among groups.

To further explore the mechanism underlying the interaction between *Gh*LAC14‐3 and *Gh*MAPKKK2, we transiently overexpressed these two genes in *N. benthamiana* leaves using the 35S promoter via *A. tumefaciens* infiltration and measured the lignin content at 48 hpi. The results showed that overexpression of *GhLAC14* alone resulted in a significant increase of 3.5‐fold in lignin content compared with control, whereas co‐expression of both genes led to an even greater increase of 5.2‐fold in lignin content (Figure 7a). We then employed the VIGS system to silence *AtMAPKKK2* in an *Arabidopsis GhLAC14‐3* overexpression line (OE‐*GhLAC14‐3*) and generated the silencing line pTRV2::*AtMAPKKK2* (Figure S6). At 14 dpi, pTRV2::*AtMAPKKK2* exhibited substantially exacerbated disease symptoms compared to the unsilenced control pTRV2::00, characterised by increased wilting and chlorosis, a higher disease index and greater fungal biomass (Figure 7b–d). In addition, upon infection with *V. dahliae*, lignin content was 1.3‐fold lower in pTRV2::*AtMAPKKK2* compared with control (Figure 7e). Collectively, these findings confirm the pivotal role of *Gh*MAPKKK2 in *Gh*LAC14‐3‐mediated lignin biosynthesis.

## 3 Discussion

Plants have evolved diverse defence mechanisms to protect their essential functions against pathogen infection (Ngou, Jones, and Ding 2022). Recent studies suggest that increasing lignin content and strengthening cell walls is an effective strategy adopted by host plants to inhibit pathogen proliferation. For example, overexpression of *AtTBL37* in *Arabidopsis* leads to enhanced acetylation of polysaccharides and thickening of the secondary cell wall, improving resistance to *Spodoptera exigua* (Sun et al. 2020). By contrast, *Arabidopsis wat1* mutants show significantly reduced secondary wall thickness, increasing their susceptibility to pathogen attacks (Denancé et al. 2013). *BnCAD5*, which encodes a cinnamyl alcohol dehydrogenase, plays a crucial role in S‐lignin biosynthesis. Increased expression of *BnCAD5* in resistant varieties of *Brassica napus* promotes S‐lignin deposition, enhancing resistance to *Sclerotinia sclerotiorum* (Höch et al. 2021). In this study, we characterised the cotton protein *Gh*LAC14‐3. It contains a laccase structural domain (Figure S1) and shows high homology to AT5G09360 (*At*LAC14), which was identified in our previous transcriptome analyses as a laccase. We therefore hypothesised that *GhLAC14‐3* may contribute to cotton resistance against VW. Indeed, RT‐qPCR analysis revealed that expression of *GhLAC14‐3* in *G. hirsutum* increased significantly upon *V. dahliae* infection (Figure 1b). We concluded that *GhLAC14‐3* is a promising disease‐resistance candidate gene that warrants further investigation. Previous studies have shown that laccases can enhance disease resistance by modulating lignin content (Blaschek et al. 2023). The G/S ratio represents the relative abundance of two lignin components: guaiacyl (G) and syringyl (S) monolignols. G monolignols are derived from coniferyl alcohol, whereas S monolignols originate from sinapyl alcohol. This ratio is a critical determinant of lignin's physical properties, influencing the ability of plant tissues to withstand mechanical stress and biological degradation (Anderson et al. 2019). For example, overexpression of *GhLAC15* in transgenic *Arabidopsis* increased cell wall lignification, total lignin content, G monolignol levels and the G/S ratio, thereby significantly improving VW resistance (Zhang et al. 2019). Similarly, *TaLAC4* expression enhanced wheat resistance to *Fusarium graminearum* by modulating G‐lignin levels in resistant cultivars (Soni et al. 2020). In this study, the knockdown of *GhLAC14‐3* in cotton compromised VW resistance (Figure 2), whereas overexpression of *GhLAC14‐3* in *Arabidopsis* enhanced VW resistance (Figure 4). Once *V. dahliae* invades the plant via the roots, it rapidly colonises and reproduces within the vascular bundle system. This fungus secretes specific toxins and enzymes that disrupt the structure and function of vascular bundle cells, thereby damaging the vascular tissues (Zhu et al. 2023). This is consistent with the higher fungal biomass detected in pTRV2::*GhLAC14‐3* plants (Figure 2e). Concurrently, plants initiate a series of defensive responses, including the production of phenolic compounds, which gradually lead to vascular browning (Zhu et al. 2023). These findings indicate that *GhLAC14‐3* plays a crucial role in disease resistance and promote further investigation into the relationship between *GhLAC14‐3* expression and lignin levels. Experimental evidence demonstrated that the silencing of *GhLAC14‐3* reduced both lignin content (Figure 3a,b) and the expression of lignin‐related genes (Figure 3c). By contrast, overexpression of *GhLAC14‐3* increased lignin content and strengthened disease resistance in *Arabidopsis*. *GhLAC14‐3* thus appears to function as a positive regulator of VW resistance through lignin accumulation. However, it remains unclear whether and how laccase participates in the intrinsic regulatory network that governs disease resistance mechanisms, and this possibility requires further investigation.

Kinase‐mediated phosphorylation modulates enzyme activity in various plant systems, enhancing disease resistance responses. Multiple potential phosphorylation sites were identified within *Gh*LAC14‐3 (Figure S1c); however, the specific kinases responsible for phosphorylating *Gh*LAC14‐3 and the exact phosphorylation sites remain to be determined. We found that *Gh*LAC14‐3 and *Gh*MAPKKK2 are localised to the cell membrane (Figures 1c and 5b; S4C), and their interactions were verified through Y2H, BiFC and LCI assays (Figure 5). Further analyses revealed that the co‐expression of *GhLAC14‐3* and *GhMAPKKK2* significantly increased lignin content (Figure 7a), whereas silencing of *AtMAPKKK2* in *GhLAC14‐3*‐overexpressing *Arabidopsis* lines resulted in reduced lignin content and disease resistance (Figure 7b–e). MAPKs are essential for transducing various extracellular stimuli in plants, particularly during biotic stress, relaying signals from upstream receptors to downstream targets through phosphorylation (Majeed et al. 2022). Pathogen infection disrupts the MAPK phosphorylation pathway in *Arabidopsis* by promoting *AtMPK1* expression, ultimately inactivating transcription factors that typically repress lignin biosynthesis genes (Lin et al. 2022). Similarly, *OsMPK5* is activated by diverse stress stimuli and interacts with OsCPK18 to modulate rice immunity through phosphorylation (Li, Zhang, Wu, et al. 2022). Overexpression of *GhNTF6* enhances resistance to *V. dahliae* in *Arabidopsis*, with mutations at Thr195 and Thr197—which disrupt its phosphorylation—reducing plant resistance to *V. dahliae* (Zhou et al. 2022). Based on our results, we speculate that *GhMAPKKK2* may be activated upon *V. dahliae* infection in cotton, leading to *Gh*LAC14‐3 phosphorylation, which in turn facilitates lignin synthesis and enhances resistance. However, the phosphorylation of *Gh*LAC14‐3 by *Gh*MAPKKK2 has yet to be confirmed. Additionally, it is important to identify critical phosphorylation sites within the kinase and to perform mutagenesis to assess whether modified versions retain the ability to phosphorylate *Gh*LAC14‐3. Further research is warranted to determine whether the interaction between *Gh*LAC14‐3 and *Gh*MAPKKK2 synergistically contributes to disease resistance in cotton.

MAPK kinases are pivotal factors that mediate diverse plant defence responses against pathogens (Meng and Zhang 2013). The activation of SIPK and WIPK mediated by *Nt*MEK2 triggers hypersensitive response (HR)‐like cell death, thereby strengthening plant defence mechanisms (Jin et al. 2003). A recent report demonstrated that *Os*MPK1 interacts with *Os*MEK2 and leads to a reactive oxygen species (ROS) burst, enhancing rice resistance against *Magnaporthe oryzae* (Dangol et al. 2021). These studies highlight how MAPK kinases can enhance plant disease resistance through various mechanisms in response to stress signals. Here, we found that overexpression of *GhMAPKKK2* in *Arabidopsis* increased its resistance against *V. dahliae* (Figure 6). Additional experiments revealed significant upregulation of multiple resistance‐related genes (Figure S5e), suggesting the conservation of MAPK functions across cotton and other plant species. We hypothesise that pathogen‐triggered activation of *GhMAPKKK2* initiates various resistance pathways via a downstream signalling cascade whose details require further exploration. Future research will focus on the interactions between *Gh*LAC14‐3 and *Gh*MAPKKK2 in disease resistance mechanisms.

**FIGURE 8:**
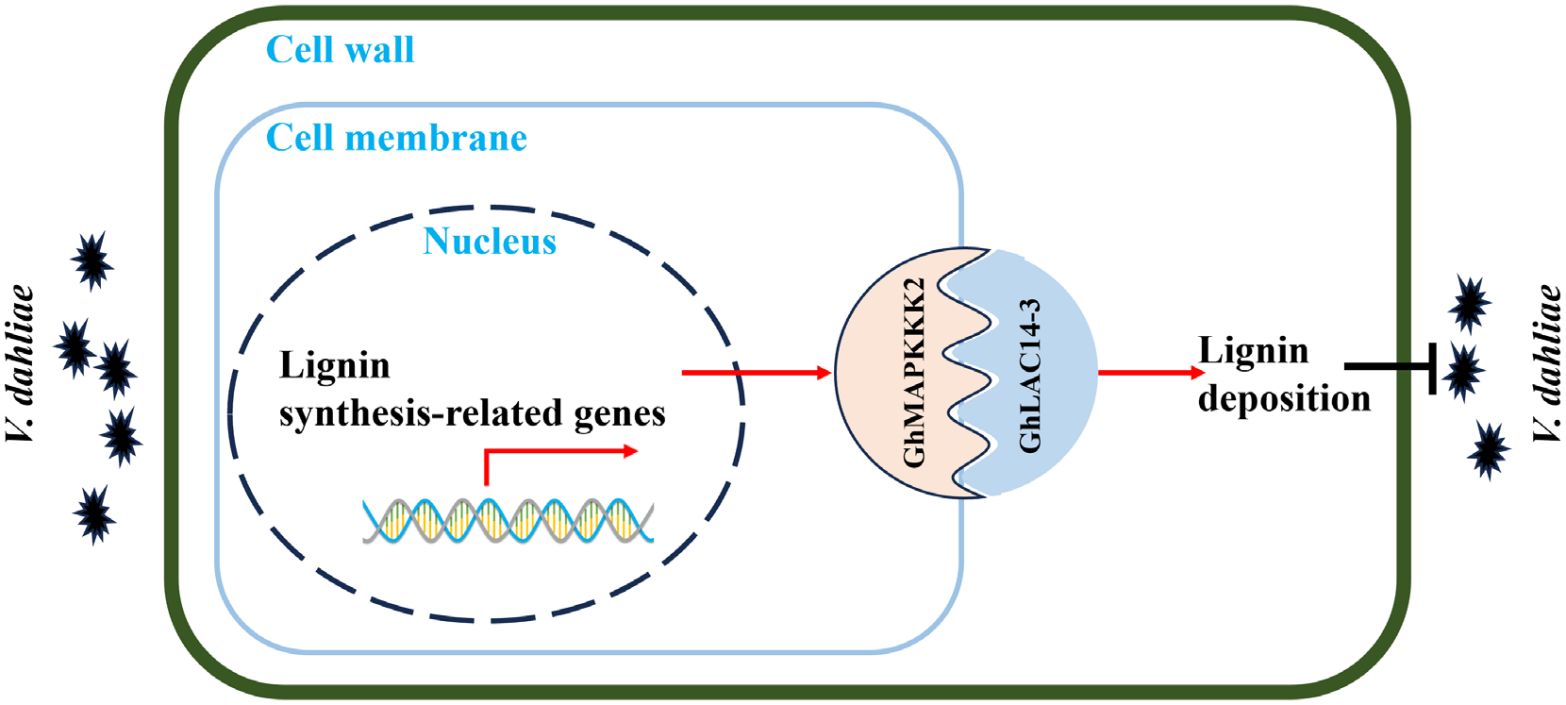
Working model of *GhLAC14‐3‐*mediated resistance against *Verticillium dahliae* infection through enhanced lignin metabolism. Upon *V. dahliae* infection, cotton initiates a defence response by significantly upregulating genes intricately involved in lignin synthesis, leading to a marked increase in the production of lignin monomers. These monomers are then actively transported from the cytoplasm to the intercellular spaces, where they are oxidised through the coordinated action of laccase *Gh*LAC14‐3 and *Gh*MAPKKK2. This initiates a series of reactions that culminate in lignin formation and lignification of the cell wall, ultimately enhancing resistance against *V. dahliae* in cotton.

In conclusion, our study identifies *Gh*LAC14‐3 as a critical positive regulator of cotton resistance to *V. dahliae*. Upon *V. dahliae* infection, the expression of *GhLAC14‐3* is rapidly upregulated, which in turn activates the transcription of lignin biosynthesis‐related genes and promotes lignin deposition. This increased lignin accumulation strengthens the cell wall, effectively hindering fungal hyphal penetration and enhancing tolerance to VW in cotton. Furthermore, our findings suggest that *Gh*MAPKKK2 interacts with *Gh*LAC14‐3 to enhance lignin production, potentially through phosphorylation, thereby enhancing its activity and VW tolerance. *Gh*MAPKKK2 may also be involved in regulating the MAPK signalling cascade, which could activate the expression of additional disease‐resistance genes, further amplifying the plant immune response (Figure 8). Together, these results underscore the synergistic roles of *GhLAC14‐3* and *GhMAPKKK2* in promoting lignin biosynthesis and strengthening cotton's resistance to *V. dahliae*. This work provides new insights into the molecular mechanisms underlying VW resistance and identifies potential targets for breeding disease‐resistant cotton cultivars.

## 4 Experimental Procedures

### 4.1 Plant Materials and Growth Conditions

The upland cotton variety Xinluzao35 was cultivated in a controlled phytotron environment set at 28°C, with 75% relative humidity, a light intensity of 400 μmol m^−2^ s^−1^, and a 16 h light/8 h dark photoperiod. For *A. thaliana* assays, the ecotype Col‐0 served as the wild type (WT). *Arabidopsis* plants were grown under similar conditions but at 21°C and 65% relative humidity. To generate heterologous expression lines for *GhLAC14‐3* and *GhMAPKKK2*, the full‐length CDSs of these genes were individually cloned into the pYBA1132 vector. The recombinant constructs were introduced into *Arabidopsis* using *A. tumefaciens* ‐mediated transformation using the floral dip method (Clough and Bent 1998). Transgenic lines were selected on Murashige and Skoog (MS) solid medium lacking sucrose and supplemented with 25 mg/L hygromycin B, followed by PCR‐based genotyping. Homozygous T_3_ lines were used for subsequent experiments. Surface‐sterilised *Arabidopsis* seeds were stratified for 3 days, germinated on MS medium for 12 days, and subsequently transferred to soil pots in growth chambers maintained at 21°C under identical light conditions. Similarly, *N. benthamiana* plants were cultivated in the same chamber. The *V. dahliae* strain Vd991 was cultured at 25°C on potato dextrose agar (200 g/L potato, 20 g/L dextrose, 15 g/L agar). Primer sequences used in this study are provided in Table S1.

### 4.2 Protein Domain and Phylogenetic Analysis of *Gh*LAC14‐3

The *AT5G09360* gene contains a typical laccase structural domain. The gene structure of *GhLAC14‐3* (XM_016843176.2) was visualised using GSDS2.0 (gsds.cbi.pku.edu.cn/). The physicochemical properties of the *Gh*LAC14‐3 protein were determined using the ExPASy‐ProtScale tool (https://web.expasy.org/protscale/). Secondary and tertiary structure predictions were obtained using PSIPRED (http://bioinf.cs.ucl.ac.uk/psipred/) and SWISS‐MODEL (https://swissmodel.expasy.org/), respectively. Protein homologues of *Gh*LAC14‐3 from various species were identified by BLAST searches of the NCBI database, and these were used to construct a phylogenetic tree of LAC proteins with iTOL (https://itol.embl.de/) and MEGA 11 (v. 11.0.13) software.

### 4.3 Subcellular Localisation

The full‐length CDSs of *GhLAC14‐3* and *GhMAPKKK2* were amplified and inserted into the pYBA1132‐GFP vector to produce the pYBA1132‐*GhLAC14‐3*‐GFP and pYBA1132‐*GhMAPKKK2*‐GFP constructs. The sequences of the primers used are provided in Table S1. These recombinant plasmids, along with an empty vector control, were introduced into *A. tumefaciens* GV3101. These constructs were co‐expressed in the leaves of *N. benthamiana* alongside either the nuclear localisation marker H2B‐mCherry or the membrane localisation marker PIP2A‐mCherry. Fluorescence signals for GFP (excitation at 488 nm, emission at 525 nm) and mCherry (excitation at 561 nm, emission at 595 nm) were analysed 3 days post‐infiltration using an LSM980 confocal laser‐scanning microscope (Zeiss).

### 4.4 Preparation and Inoculation of *V. dahliae* Pathogen

The preserved *V. dahliae* Vd991 strain was cultured in a complete medium consisting of 1 g/L yeast extract, 10 g/L dextrose, 0.2% NaNO_3_, 0.131% K_2_HPO_4_, 0.05% MgSO_4_, 0.05% KCl and 0.002% FeSO_4_ at 25°C with shaking at 200 rpm for 3–4 days. The spores were then harvested, washed, and resuspended in distilled water to a final concentration of 2 × 10^7^ cfu/mL. For inoculation, cotton seedlings at the two‐true‐leaf stage or 21‐day‐old *A. thaliana* plants had their roots immersed in the spore suspension for 5 min before being transplanted into fresh soil. Roots from three individual cotton seedlings were collected at 0, 0.5, 1, 2, 4 and 8 hpi with Vd991 for further analysis.

### 4.5 RT‐PCR and RT‐qPCR

Total RNA was extracted using the RNAsecure Plant Kit (Tiangen Bio). Subsequently, 1 μg of total RNA was reverse transcribed into cDNA using the HiScriptIII RT SuperMix (Vazyme). Gene expression levels were quantified by RT‐qPCR using the BioGold SYBR qPCR Master Mix (Biogene) on an OptiFlex optical system. Each analysis was performed with three biological replicates, and relative gene expression was calculated using the $2^{-\Delta \Delta C_{t}}$ method (Livak and Schmittgen 2001). Additionally, gene expression was assessed by RT‐PCR using Rapid Taq Master Mix (Vazyme), with *GhUBQ7* (LOC107912293) or *Actin2* (AT3G18780) as endogenous controls for cotton and *Arabidopsis*, respectively. Primer sequences for RT‐PCR and RT‐qPCR are provided in Table S1. Reverse transcription system and qPCR procedure are shown in Tables S2 and S3. RT‐qPCR system and RT‐qPCR procedure are shown in Tables S4 and S5.

### 4.6 VIGS Assay

A tobacco rattle virus (TRV)‐based VIGS system was employed for the functional analysis of *GhLAC14‐3*. The silencing fragment of *GhLAC14‐3* was designed using the SGN‐VIGS online tool (https://vigs.solgenomics.net/) and PCR amplified from Xinluzao 35 seedling cDNA. This fragment was then inserted into the pTRV2 vector to construct the pTRV2::*GhLAC14‐3* plasmid. pTRV2::*GhLAC14‐3*, along with the negative control pTRV2::00 and the positive control pTRV2::*GhCLA1* plasmids, was separately introduced into *A. tumefaciens* GV3101. The GV3101 cultures (OD_600_ = 1.0–1.2) containing the pTRV2 plasmids were mixed with cultures harbouring the pTRV1 vector at a 1:1 (vol/vol) ratio and co‐infiltrated into the cotyledons of 8‐day‐old Xinluzao 35 seedlings. For each VIGS treatment, more than 10 seedlings were used. Approximately 14 dpi, when the pTRV2::*GhCLA1*‐treated plants displayed a distinct albino phenotype, roots from the treated seedlings in each group were collected for RNA extraction. The silencing efficiency of *GhLAC14‐3* was evaluated by RT‐qPCR. Primer sequences for PCR and RT‐qPCR are provided in Table S1. The PCR system and procedure are shown in Tables S6 and S7. The gene names and numbers used in this paper are listed in Table S8.

### 4.7 Disease Severity Observation and Quantification

The severity of plant disease was assessed by calculating a disease index, accounting for leaf wilting and vascular discolouration in the stems. For cotton seedlings treated with VIGS and *Arabidopsis* plants, disease symptoms were rated on a scale of 0 to 4, and the disease index was calculated based on previously published methods (Wang et al. 2020). The disease index was determined using the formula: disease index (%) = [(∑ disease grades × number of infected plants)/(total plants examined × 4)] × 100. To quantify fungal biomass, the method described by Li, Zhou, et al. (2021) was followed. Roots from inoculated plants were collected, and genomic DNA was extracted using the CTAB method. Relative fungal biomass was then quantified by qPCR, using the fungus‐specific primer ITS‐F (targeting the internal transcribed spacer [ITS] region of ribosomal DNA) and the *V. dahliae*‐specific reverse primer STVe1‐R (Xu et al. 2018). *Actin2* and *GhUBQ7* served as endogenous reference genes for *Arabidopsis* and cotton, respectively. All primer sequences are provided in Table S1. PCRs with these primers were conducted using an annealing temperature of 60°C and an extension time of 20 s.

### 4.8 Histochemical Staining and Quantification of Lignin

Cross sections from the base of stem internodes were prepared by hand‐cutting for histochemical staining. Total lignin deposition was visualised using a resorcinol staining kit (Solarbio). Stem sections approximately 0.2 mm thick were immersed in a 1% ethanol solution of resorcinol for 10 min. After staining, the slices were mounted on slides and observed under a microscope (Thermo) to examine lignin deposition in stem tissues. For lignin quantification, approximately 5 mg of cotton stems or *N. benthamiana* leaves was collected, and the lignin content was measured using a lignin content assay kit (Solarbio), following the protocol described by (Wang et al. 2024). Absorbance was measured at 280 nm using a UV spectrophotometer (Thermo). The lignin content was calculated using the formula: lignin content (mg/g) = 2.184 × (A − A_0_)/0.005, where A is the absorbance of the experimental group, and A_0_ is the absorbance of the blank control.

### 4.9 Yeast Library Screening and Y2H Assay

The full‐length CDS of *GhLAC14‐3* was amplified and cloned into the pGBKT7 (BD) vector to create the BD‐*GhLAC14‐3* construct. This plasmid, along with the empty pGADT7 (AD) vector, was transformed into the yeast strain AH109. Transformed cells were plated onto synthetic defined (SD) medium lacking Leu and Trp (SD/−Trp/−Leu) as well as medium lacking Leu, Trp, His, and Ade (SD/−Trp/−Leu/−His/−Ade) for 2 days to evaluate whether *Gh*LAC14‐3 exhibited self‐activation. To identify potential interacting proteins, the pGBKT7‐*GhLAC14‐3* construct was co‐transformed with a yeast library plasmid into AH109 and plated on SD/−Trp/−Leu medium. After approximately 3 days of growth, a single clone was transferred to SD/−Trp/−Leu/−His/−Ade medium for an additional 5 days. Positive clones were selected, their inserts amplified by PCR and sequenced. The resulting sequence was analysed using BLASTP against the NCBI database, identifying the disease‐resistance protein *Gh*MAPKKK2 (XM_016855464.2). The full‐length CDS of *GhMAPKKK2* was subsequently cloned into the pGADT7 vector to generate AD‐*GhMAPKKK2*. Yeast strain AH109 was co‐transformed with BD‐*GhLAC14‐3* and AD‐*GhMAPKKK2*. The growth of transformed cells was monitored on SD/−Trp/−Leu and SD/−Trp/−Leu/−His/−Ade media. Further confirmation was conducted by assessing growth on SD/−Trp/−Leu/−His/−Ade medium supplemented with Xα‐Gal.

### 4.10 BiFC and LCI Assays

The full‐length CDS of *GhLAC14‐3* was amplified and inserted into the pIB‐YN155 vector to generate the *GhLAC14‐3*–nYFP construct. Similarly, the CDS of *GhMAPKKK2* was amplified and cloned into the pIB‐VC156 vector to create the *GhMAPKKK2*–cYFP construct. Primer sequences used for cloning are listed in Table S1. BiFC assays were performed following established protocols (Xing et al. 2024). Different combinations of nYFP‐ and cYFP‐fused proteins were transiently expressed in *N. benthamiana* leaves through *A. tumefaciens* ‐mediated transformation. Three days post‐infiltration, fluorescence signals for yellow fluorescent protein (YFP; excitation at 514 nm and emission at 549 nm) were detected using a laser scanning confocal microscope (LSM980; Zeiss).

The full‐length CDS of *GhLAC14‐3* was also amplified and inserted into the pCAMBIA1300‐nLuc vector to generate the *GhLAC14‐3*–nLUC construct. Similarly, the CDS of GhMAPKKK2 was amplified and cloned into pCAMBIA1300‐cLuc, resulting in the *GhMAPKKK2*–cLUC construct. The resulting GhLAC14‐3–nLUC and *GhMAPKKK2*–cLUC constructs were introduced into *A. tumefaciens* GV3101 and transiently expressed in *N. benthamiana* leaves using *A. tumefaciens* ‐mediated transformation. Fluorescence signals were captured 2 days post‐transformation with a plant live imaging system (NightSHADE LB 985) to visualise the protein–protein interactions.

### 4.11 Statistical Analysis

All experiments were conducted at least three times, and quantitative data are presented as the mean ± SE or *SEM*. Statistical significance was determined using Student's *t*‐ test, with significance indicated by asterisks (*p < 0.05, **p < 0.01, and ***p < 0.001). For multiple comparisons, one‐way ANOVA followed by Duncan's test was used, with significant differences represented by different letters at *p* < 0.05.

## Author Contributions

**Guanfu Cheng:** writing – original draft, software, data curation, formal analysis, validation, conceptualization, methodology, investigation. **Chuanzong Li:** conceptualization, methodology, software, validation, writing – original draft. **Guoshuai Zhang:** investigation, formal analysis. **W. G. Dilantha Fernando:** writing – review and editing. **Yanqing Bi:** validation, investigation. **Jianfeng Lei:** methodology, software. **Peihong Dai:** supervision, visualization, writing – review and editing. **Xiaofeng Su:** writing – review and editing, supervision, formal analysis, validation, investigation. **Yue Li:** funding acquisition, project administration, resources, writing – review and editing, supervision.

## Conflicts of Interest

The authors declare no conflicts of interest.

## Supporting information


**Figure S1.** Multiple sequence alignment of *Gh*LAC14‐3 with other *Gh*LAC14 proteins and phylogenetic tree of *Gh*LAC14‐3 homologues.

**Figure S2.** Schematic representation of the *GhLAC14‐3* overexpression construct, driven by the 35S constitutive promoter upstream of the *GhLAC14‐3* coding sequence.

**Figure S3.** Expression of genes related to lignin synthesis in *GhLAC14‐3*‐overexpressing *Arabidopsis*.

**Figure S4.** Structural features and phylogeny of *Gh*MAPKKK2.

**Figure S5.** Overexpression of *GhMAPKKK2* enhances resistance to *Verticillium dahliae* in *Arabidopsis*.

**Figure S6.** Relative expression levels of *AtMAPKKK2* in *Arabidopsis*.

**Table S1.** Primers used in this study.

**Table S2.** Reverse transcription system.

**Table S3.** RT‐PCR procedure.

**Table S4.** RT‐qPCR system.

**Table S5.** RT‐qPCR procedure.

**Table S6.** PCR system.

**Table S7.** PCR procedure.

**Table S8.** Gene name and number.

## Data Availability

The data supporting the findings of this study are available from the corresponding author upon request for academic or scientific purposes, subject to ethical considerations and confidentiality agreements.
