# Supplementary figures and images for: A Cotton Laccase Confers Disease Resistance Against Verticillium dahliae by Promoting Cell Wall Lignification
Source: Mol Plant Pathol. 2025 Jul 14;26(7):e70125. doi: 10.1111/mpp.70125 (PMC12257636; doi:10.1111/mpp.70125)

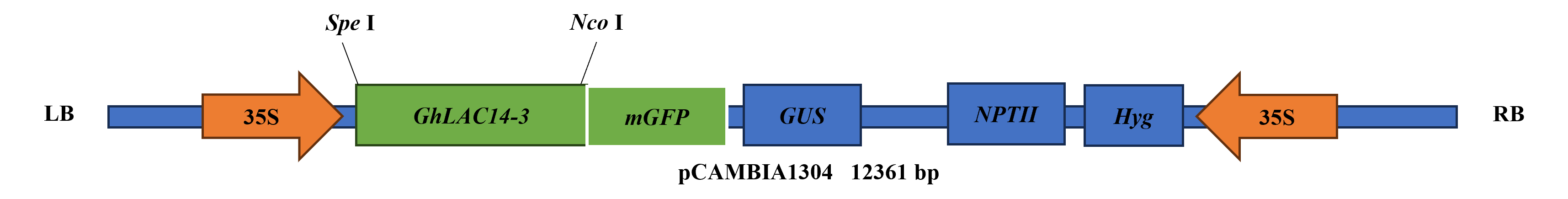

Supplement: Supplementary file 2 — Figure S2. Schematic representation of the GhLAC14‐3 overexpression construct, driven by the 35S constitutive promoter upstream of the GhLAC14‐3 coding sequence. [file MPP-26-e70125-s010.png]

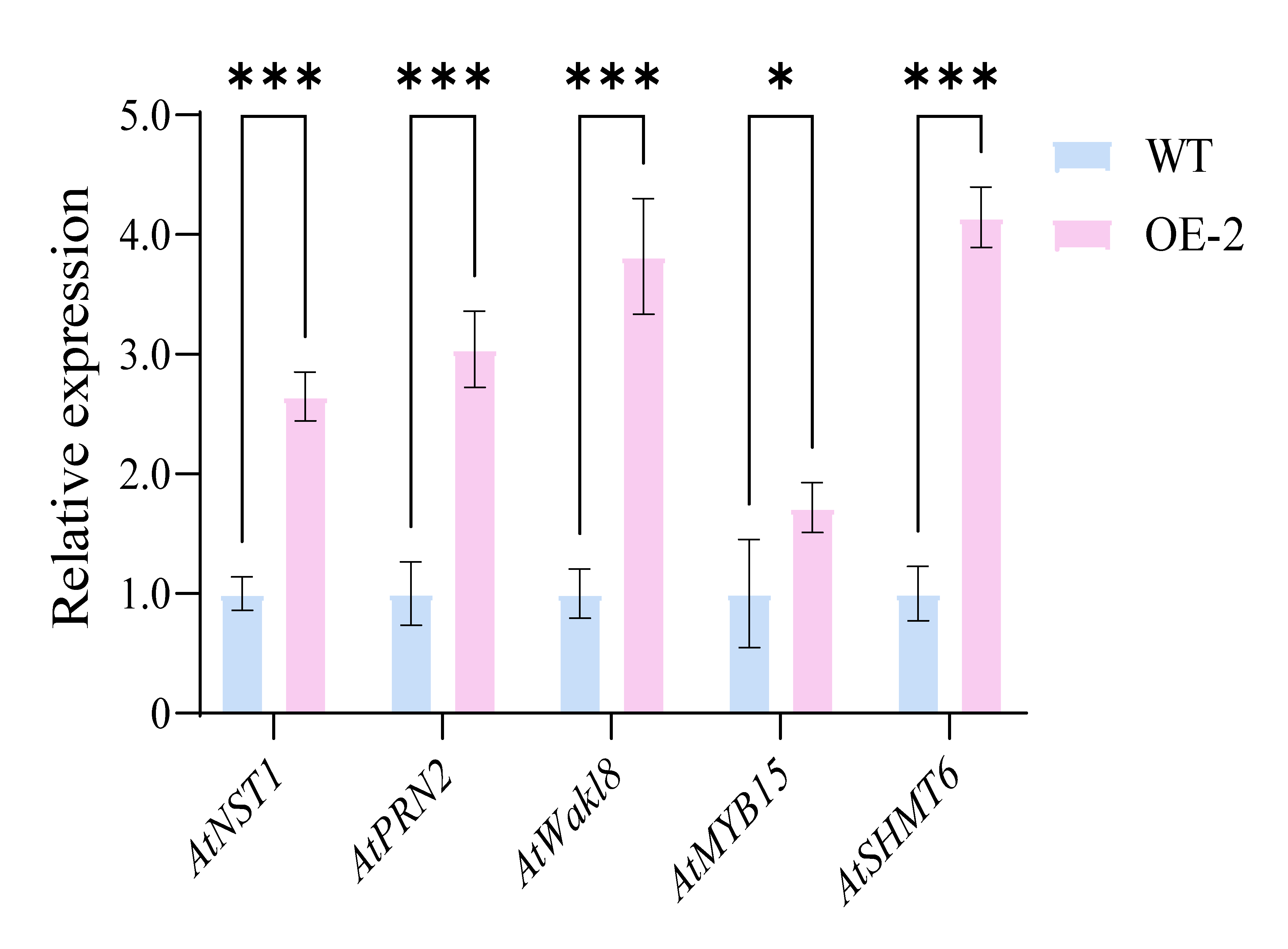

Supplement: Supplementary file 3 — Figure S3. Expression of genes related to lignin synthesis in GhLAC14‐3‐overexpressing Arabidopsis. [file MPP-26-e70125-s001.tif]

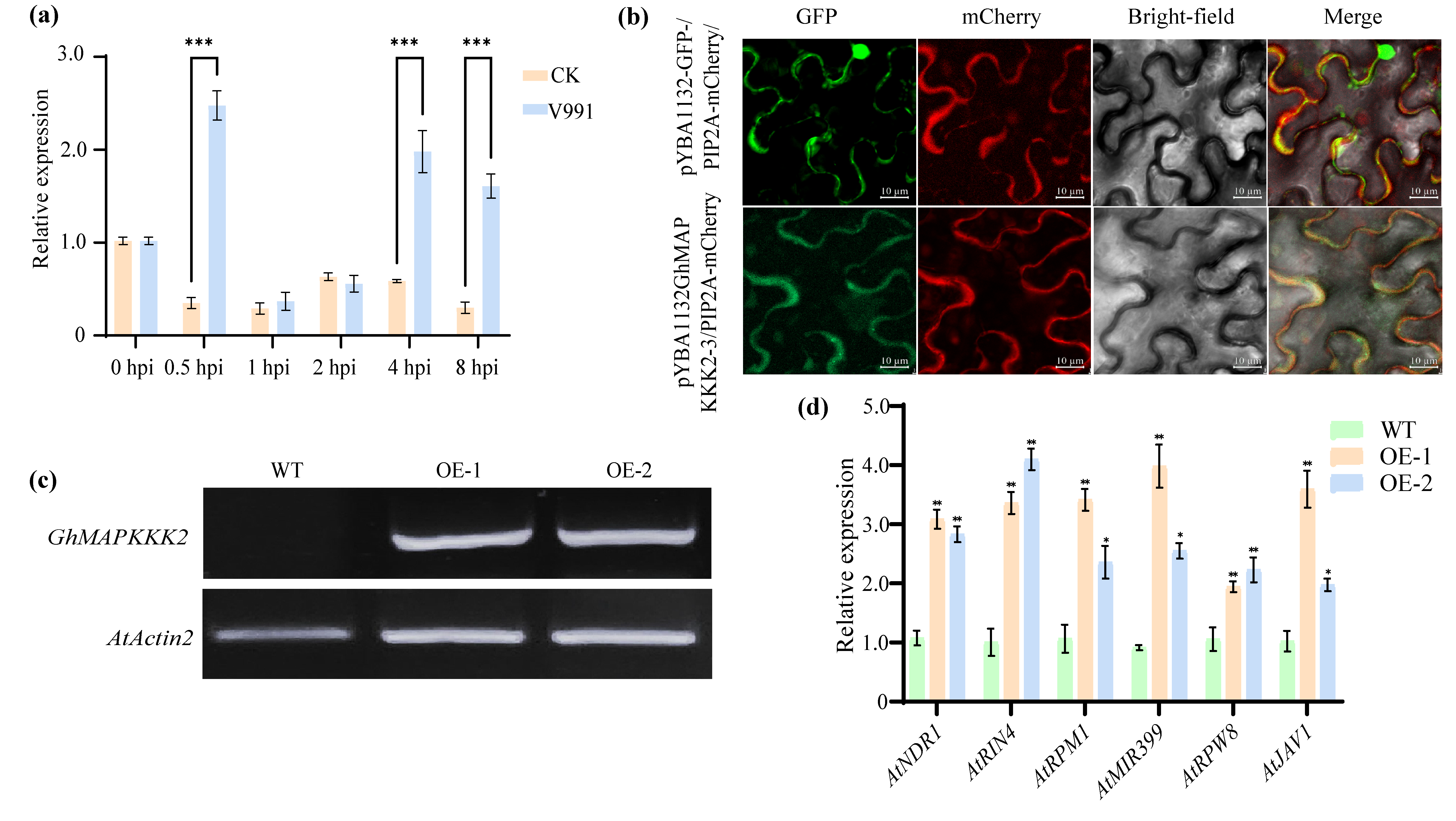

Supplement: Supplementary file 5 — Figure S5. Overexpression of GhMAPKKK2 enhances resistance to Verticillium dahliae in Arabidopsis. [file MPP-26-e70125-s008.tif]

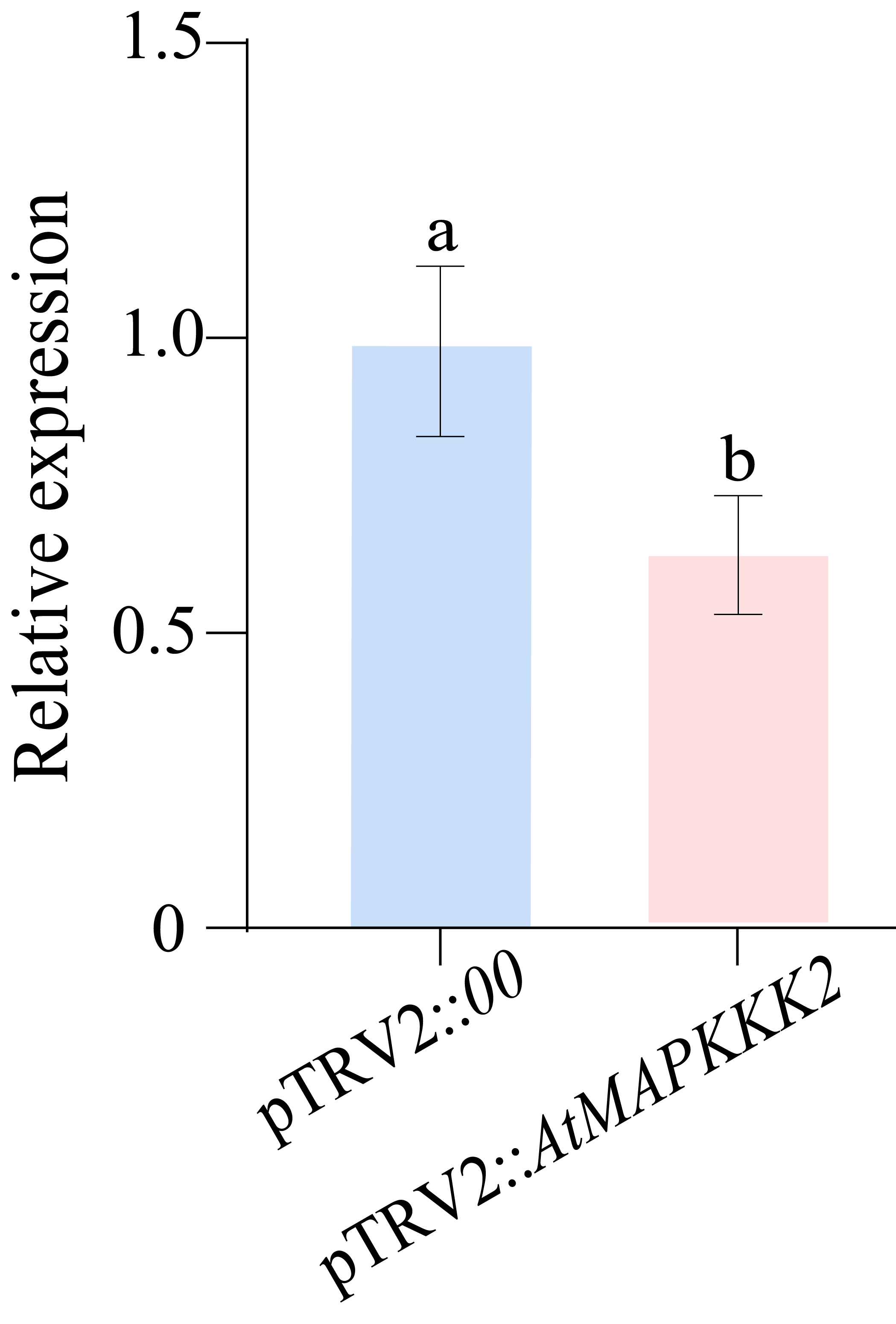

Supplement: Supplementary file 6 — Figure S6. Relative expression levels of AtMAPKKK2 in Arabidopsis. [file MPP-26-e70125-s009.png]
