# Supplementary material for: A Cotton Laccase Confers Disease Resistance Against Verticillium dahliae by Promoting Cell Wall Lignification
Source: Mol Plant Pathol. 2025 Jul 14;26(7):e70125. doi: 10.1111/mpp.70125 (PMC12257636; doi:10.1111/mpp.70125)
Supplement: Supplementary file 7 — Table S1. Primers used in this study. [file MPP-26-e70125-s006.docx]

**Table S1.** Primers used in this study.

| Primer name | Sequence (5′–3′) | Purpose |
| --- | --- | --- |
| q*GhLAC14-3*-F | CGTCCACGGTGCCTTTGT | RT-qPCR |
| q*GhLAC14-3*-R | GTCAGAAGTAGGTGCGGGAA |  |
| q*GhMAPKKK2*-F | GACACCCAGCAGCGTATTG |  |
| q*GhMAPKKK2*-R | CAAAGAGAAAACCAGATCGTC |  |
| q*GhUBQ7*-F | GAAGGCATTCCACCTGACCAAC |  |
| q*GhUBQ7*-R | CTTGACCTTCTTCTTCTTGTGCTTG |  |
| q*AtMAPKKK2*-F | GTTTCACCGGTCGGAGTTCT |  |
| q*AtMAPKKK2*-R | TGTGGAAGGGCTCTCTCGTA |  |
| q*Actin2*-F | GCACCCTGTTCTTCTTACCG |  |
| q*Actin2*-R | AACCCTCGTAGATTGGCACA |  |
| ITS-F | CCGCCGGTCCATCAGTCTCTCTGTTTATAC |  |
| STVe1-R | CGCCTGCGGGACTCCGATGCGAGCTGTAAC |  |
| q*GhPAL*-F | GGGAGATACTTGAAGCGAT |  |
| q*GhPAL*-R | TTGGAGTTATGTCTGCCTG |  |
| q*GhCCR1*-F | GACCTTGTGGCGATAACC |  |
| q*GhCCR1*-R | GCTAATGCGACATCTCTGA |  |
| q*GhCCoAOMT1*-F | CACCTGGGTCACAATCCCTTAC |  |
| q*GhCCoAOMT1*-R | CCAACTGTGGCACGGCAAT |  |
| q*GhC4H1*-F | GGACCCACCAGTTTATTG |  |
| q*GhC4H1*-R | ACCAGATTACGCTGTCCC |  |
| q*GhCOMT*-F | AGATCGAATGCTACGCCTGT |  |
| q*GhCOMT*-R | GCAACAAAAGGAGAGGTGCA |  |
| q*AtNST1*-F | TGAGGTCGTGAGTATTATAGG |  |
| q*AtNST1*-R | TCTGAGATGATGTCGTCTTC |  |
| q*AtPRN2*-F | GAGTGAGCAGAAGTTGTTAG |  |
| q*AtPRN2*-R | TGAAGTCTTGGTGAGTGATT |  |
| q*AtWakl8*-F | CAACAACAAGGCGTTCAT |  |
| q*AtWakl8*-R | AGAAGAGGTGGTATAGTCATC |  |
| q*AtMYB15*-F | GTAGACTTAGGTGGATGAACT |  |
| q*AtMYB15*-R | TCGCTGACCATCTATTGC |  |
| q*AtSHMT6*-F | CGTCCTTCGTCTAATCCTAT |  |
| q*AtSHMT6*-R | TCATCGTCCTCCTCAACA |  |
| q*AtNDR1*-F | CAAGAGCAACAACGGTTAA |  |
| q*AtNDR1*-R | ACGGATACACCACACCTA |  |
| q*AtRIN4*-F | GGTGACGGTAACGGTAAC |  |
| q*AtRIN4*-R | CTAGTGGTGATGCTATGTATTC |  |
| q*AtRPM1*-F | GTGCGTTTCATTTCCCGAGG |  |
| q*AtRPM1*-R | TCCCTTGGGTGCATCAATCC |  |
| q*AtMIR399*-F | AAGATCACCATTGGCAGAG |  |
| q*AtMIR399*-R | AAGAGAATTACCGGGCAAAT |  |
| q*AtRPW8*-F | GTCTCGTTGATGCTTATGC |  |
| q*AtRPW8*-R | GCCATTGATTGACTTGAACA |  |
| q*AtJAV1*-F | CGAGTGGTCTCAGTTCTAC |  |
| q*AtJAV1*-R | AGTCTATGGAGGTGGTATCA |  |
| 1132-*GhLAC14-3*-F | cgctctagaactagtggatccATGGGTTCTGAAAAGCAAGGG | Localization |
| 1132-*GhLAC14-3*-R | gataagcttgatatcgaattcTTAAGAACTAGTGGTGAGATATCGTAGA |  |
| 1132-*GhMAPKKK2*-F | cgctctagaactagtggatccATGGACTGGACACGTGGCAG |  |
| 1132-*GhMAPKKK2*-R | gataagcttgatatcgaattcCTGCAGTTAAGGATGTGGGTTTG |  |
| 1304-*GhLAC14-3*-F | acgggggactcttgaccatggATGGGTTCTGAAAAGCAAGGG | Overexpression |
| 1304-*GhLAC14-3*-R | aagttcttctcctttactagtTTAAGAACTAGTGGTGAGATATCGTAGA |  |
| 1304-*GhMAPKKK2*-F | acgggggactcttgaccatggATGGACTGGACACGTGGCA |  |
| 1304-*GhMAPKKK2*-R | aagttcttctcctttactagtAGGATGTGGGTTTGGATCCAT |  |
| pGBKT7-*GhLAC14-3*-F | atggccatggaggccgaattcATGGGTTCTGAAAAGCAAGGG | Y2H assays |
| pGBKT7-*GhLAC14-3*-R | ccgctgcaggtcgacggatccTTAAGAACTAGTGGTGAGATATCGTAGA |  |
| pGADT7-*GhMAPKKK2*-F | gccatggaggccagtgaattcATGGACTGGACACGTGGCA |  |
| pGADT7-*GhMAPKKK2*-R | cagctcgagctcgatggatccAGGATGTGGGTTTGGATCCAT |  |
| pYFPNE-*GhLAC14-3*-F | tggcgcgccactagtggatccATGGGTTCTGAAAAGCAAGGG | BiFC assays |
| pYFPNE-*GhLAC14-3*-R | agcggtaccctcgaggtcgacTTAAGAACTAGTGGTGAGATATCGTAGA |  |
| pYFPCE-*GhMAPKKK2*-F | tggcgcgccactagtggatccATGGACTGGACACGTGGCA |  |
| pYFPCE-*GhMAPKKK2*-R | agcggtaccctcgaggtcgacAGGATGTGGGTTTGGATCCAT |  |
| pLUCN-*GhLAC14-3*-F | cgggggacgagctcggtaccATGGGTTCTGAAAAGCAAGGG | LCI assays |
| pLUCN-*GhLAC14-3*-R | acgagatctggtcgacTTAAGAACTAGTGGTGAGATATCGTAGA |  |
| pLUCC-*GhMAPKKK2*-F | acgcgtcccggggcggtaccATGGACTGGACACGTGGCA |  |
| pLUCC-*GhMAPKKK2*-R | agctctgcaggtcgacAGGATGTGGGTTTGGATCCAT |  |
