# Supplementary material for: A Cotton Laccase Confers Disease Resistance Against Verticillium dahliae by Promoting Cell Wall Lignification
Source: Mol Plant Pathol. 2025 Jul 14;26(7):e70125. doi: 10.1111/mpp.70125 (PMC12257636; doi:10.1111/mpp.70125)
Supplement: Supplementary file 8 — Table S2. Reverse transcription system. [file MPP-26-e70125-s004.docx]

**Table S2** Reverse transcription system

| Reagent | Usage amount/μL |
| --- | --- |
| Total RNA | 2-4 |
| 5×All-In-One RT MasterMix with AccuRT | 4 |
| Nuclease-free H_2_O | 12-14 |
