# Supplementary material for: A Cotton Laccase Confers Disease Resistance Against Verticillium dahliae by Promoting Cell Wall Lignification
Source: Mol Plant Pathol. 2025 Jul 14;26(7):e70125. doi: 10.1111/mpp.70125 (PMC12257636; doi:10.1111/mpp.70125)
Supplement: Supplementary file 9 — Table S3. RT‐PCR procedure. [file MPP-26-e70125-s014.docx]

**Table S3** RT-PCR reaction procedure

| Temperature/℃ | Time/min |
| --- | --- |
| 37 | 15 |
| 60 | 10-15 |
| 95 | 3 |
| 4 | ∞ |
