# Supplementary material for: A Cotton Laccase Confers Disease Resistance Against Verticillium dahliae by Promoting Cell Wall Lignification
Source: Mol Plant Pathol. 2025 Jul 14;26(7):e70125. doi: 10.1111/mpp.70125 (PMC12257636; doi:10.1111/mpp.70125)
Supplement: Supplementary file 10 — Table S4. RT‐qPCR system. [file MPP-26-e70125-s013.docx]

**Table S4** RT-qPCR reaction system.

| Reagent | Usage amount/μL |
| --- | --- |
| Template DNA | 1-2 |
| Forward Primer | 0.5 |
| Reverse Primer | 0.5 |
| BlasTaqTM 2×qPCR MM | 10 |
| Nuclease-free H_2_O | 7-8 |
| Total Volume | 20 |
