# Supplementary material for: A Cotton Laccase Confers Disease Resistance Against Verticillium dahliae by Promoting Cell Wall Lignification
Source: Mol Plant Pathol. 2025 Jul 14;26(7):e70125. doi: 10.1111/mpp.70125 (PMC12257636; doi:10.1111/mpp.70125)
Supplement: Supplementary file 11 — Table S5. RT‐qPCR procedure. [file MPP-26-e70125-s012.docx]

**Table S5** RT-qPCR reaction procedure

| Temperature/℃ | Time/s | Number of cycles |
| --- | --- | --- |
| 95 | 30 | 1 |
| 95 | 5 | 40 |
| 60 | 20 | 40 |
