# Supplementary material for: A Cotton Laccase Confers Disease Resistance Against Verticillium dahliae by Promoting Cell Wall Lignification
Source: Mol Plant Pathol. 2025 Jul 14;26(7):e70125. doi: 10.1111/mpp.70125 (PMC12257636; doi:10.1111/mpp.70125)
Supplement: Supplementary file 12 — Table S6. PCR system. [file MPP-26-e70125-s005.docx]

**Table S6** PCR reaction system.

| Reagent | Usage amount/μL |
| --- | --- |
| Template DNA | 2 |
| Forward Primer | 2 |
| Reverse Primer | 2 |
| Phusion mix | 10 |
| ddH_2_O | 4 |
| Total Volume | 20 |
