# Supplementary material for: A Cotton Laccase Confers Disease Resistance Against Verticillium dahliae by Promoting Cell Wall Lignification
Source: Mol Plant Pathol. 2025 Jul 14;26(7):e70125. doi: 10.1111/mpp.70125 (PMC12257636; doi:10.1111/mpp.70125)
Supplement: Supplementary file 13 — Table S7. PCR procedure. [file MPP-26-e70125-s007.docx]

**Table S7** PCR reaction procedure

| Temperature | Time | Cycle |
| --- | --- | --- |
| 98℃ | 30 s | 1 |
| 98℃ | 10 s | 35 |
| 60-62℃ | 30 s |  |
| 72℃ | 10-15 s |  |
| 72℃ | 10 min | 1 |
| 4℃ | ∞ |  |
