# Supplementary material for: A Cotton Laccase Confers Disease Resistance Against Verticillium dahliae by Promoting Cell Wall Lignification
Source: Mol Plant Pathol. 2025 Jul 14;26(7):e70125. doi: 10.1111/mpp.70125 (PMC12257636; doi:10.1111/mpp.70125)
Supplement: Supplementary file 14 — Table S8. Gene name and number. [file MPP-26-e70125-s003.docx]

**Table S8** Gene name and number.

| Gene | Gene name | number |
| --- | --- | --- |
| *GhPAL* | Gossypium hirsutum phenylalanine ammonia-lyase | LOC107897711 |
| *GhCCR1* | Gossypium hirsutum cinnamoyl-CoA reductase 1 | LOC107958455 |
| *GhCCoAOMT1* | Gossypium hirsutum caffeoyl-CoA O-methyltransferase | LOC107938099 |
| *GhC4H1* | Gossypium hirsutum trans-cinnamate 4-monooxygenase | LOC107936045 |
| *GhCOMT* | Gossypium hirsutum caffeic acid 3-O-methyltransferase | LOC107939017 |
| *AtNST1* | Arabidopsis thaliana NAC (No Apical Meristem) domain transcriptional regulator superfamily protein (NST1) | AT2G46770 |
| *AtPRN2* | Arabidopsis thaliana RmlC-like cupins superfamily protein | AT2G43120 |
| *AtWakl8* | Arabidopsis thaliana Wall-associated kinase family protein | AT1G16260 |
| *AtMYB15* | Arabidopsis thaliana myb domain protein 15 (MYB15) | AT3G23250 |
| *AtSHMT6* | Arabidopsis thaliana serine hydroxymethyltransferase 6 (SHM6) | AT1G22020 |
| *AtNDR1* | Arabidopsis thaliana NDR1/HIN1 | AT5G36970 |
| *AtRIN4-* | Arabidopsis thaliana RPM1 interacting protein 4 (RIN4) | AT3G25070 |
| *AtRPM1* | Arabidopsis thaliana NB-ARC domain-containing disease resistance protein (RPM1) | AT3G07040 |
| *AtMIR399* | Arabidopsis thaliana Disease resistance protein (TIR-NBS-LRR class) family | AT1G64070 |
| *AtRPW8* | Arabidopsis thaliana homolog of RPW8 | AT3G50450 |
| *AtJAV1* | Arabidopsis thaliana VQ motif-containing protein | AT3G22160 |
